# Polymerization Reactions and Modifications of Polymers by Ionizing Radiation

**DOI:** 10.3390/polym12122877

**Published:** 2020-11-30

**Authors:** Aiysha Ashfaq, Marie-Claude Clochard, Xavier Coqueret, Clelia Dispenza, Mark S. Driscoll, Piotr Ulański, Mohamad Al-Sheikhly

**Affiliations:** 1 Department of Chemistry and Biochemistry, University of Maryland, College Park, MD 20742, USA; aiysha@umd.edu; 2 Laboratoire des Solides Irradiés, CEA/DRF/IRAMIS-CNRS- Ecole Polytechnique UMR 7642, Institut Polytechnique de Paris, 91128 Palaiseau, France; marie-claude.clochard@polytechnique.edu; 3 Institut de Chimie Moléculaire de Reims, CNRS UMR 7312, Université de Reims Champagne-Ardenne, BP 1039, 51687 Reims CEDEX 2, France; xavier.coqueret@univ-reims.fr; 4 Dipartimento di Ingegneria, Università degli Studi di Palermo, Viale delle Scienze 6, 90128 Palermo, Italy; clelia.dispenza@unipa.it; 5 Istituto di BioFisica, Consiglio Nazionale delle Ricerche, Via U. La Malfa 153, 90146 Palermo, Italy; 6 Department of Chemistry, State University of New York College of Environmental Science and Forestry, Syracuse, NY 13210, USA; mdriscol@esf.edu; 7 UV/EB Technology Center, State University of New York College of Environmental Science and Forestry, Syracuse, NY 13210, USA; 8 Institute of Applied Radiation Chemistry, Faculty of Chemistry, Lodz University of Technology, Wroblewskiego 15, 93-590 Lodz, Poland; piotr.ulanski@p.lodz.pl; 9 Department of Materials Science and Engineering, University of Maryland, College Park, MD 20742, USA

**Keywords:** radiation induced polymerization, ionizing radiation, radiation synthesis nanogels, radiation induced grafting, radiation of natural polymers

## Abstract

Ionizing radiation has become the most effective way to modify natural and synthetic polymers through crosslinking, degradation, and graft polymerization. This review will include an in-depth analysis of radiation chemistry mechanisms and the kinetics of the radiation-induced C-centered free radical, anion, and cation polymerization, and grafting. It also presents sections on radiation modifications of synthetic and natural polymers. For decades, low linear energy transfer (LLET) ionizing radiation, such as gamma rays, X-rays, and up to 10 MeV electron beams, has been the primary tool to produce many products through polymerization reactions. Photons and electrons interaction with polymers display various mechanisms. While the interactions of gamma ray and X-ray photons are mainly through the photoelectric effect, Compton scattering, and pair-production, the interactions of the high-energy electrons take place through coulombic interactions. Despite the type of radiation used on materials, photons or high energy electrons, in both cases ions and electrons are produced. The interactions between electrons and monomers takes place within less than a nanosecond. Depending on the dose rate (dose is defined as the absorbed radiation energy per unit mass), the kinetic chain length of the propagation can be controlled, hence allowing for some control over the degree of polymerization. When polymers are submitted to high-energy radiation in the bulk, contrasting behaviors are observed with a dominant effect of cross-linking or chain scission, depending on the chemical nature and physical characteristics of the material. Polymers in solution are subject to indirect effects resulting from the radiolysis of the medium. Likewise, for radiation-induced polymerization, depending on the dose rate, the free radicals generated on polymer chains can undergo various reactions, such as inter/intramolecular combination or inter/intramolecular disproportionation, b-scission. These reactions lead to structural or functional polymer modifications. In the presence of oxygen, playing on irradiation dose-rates, one can favor crosslinking reactions or promotes degradations through oxidations. The competition between the crosslinking reactions of C-centered free radicals and their reactions with oxygen is described through fundamental mechanism formalisms. The fundamentals of polymerization reactions are herein presented to meet industrial needs for various polymer materials produced or degraded by irradiation. Notably, the medical and industrial applications of polymers are endless and thus it is vital to investigate the effects of sterilization dose and dose rate on various polymers and copolymers with different molecular structures and morphologies. The presence or absence of various functional groups, degree of crystallinity, irradiation temperature, etc. all greatly affect the radiation chemistry of the irradiated polymers. Over the past decade, grafting new chemical functionalities on solid polymers by radiation-induced polymerization (also called RIG for Radiation-Induced Grafting) has been widely exploited to develop innovative materials in coherence with actual societal expectations. These novel materials respond not only to health emergencies but also to carbon-free energy needs (e.g., hydrogen fuel cells, piezoelectricity, etc.) and environmental concerns with the development of numerous specific adsorbents of chemical hazards and pollutants. The modification of polymers through RIG is durable as it covalently bonds the functional monomers. As radiation penetration depths can be varied, this technique can be used to modify polymer surface or bulk. The many parameters influencing RIG that control the yield of the grafting process are discussed in this review. These include monomer reactivity, irradiation dose, solvent, presence of inhibitor of homopolymerization, grafting temperature, etc. Today, the general knowledge of RIG can be applied to any solid polymer and may predict, to some extent, the grafting location. A special focus is on how ionizing radiation sources (ion and electron beams, UVs) may be chosen or mixed to combine both solid polymer nanostructuration and RIG. LLET ionizing radiation has also been extensively used to synthesize hydrogel and nanogel for drug delivery systems and other advanced applications. In particular, nanogels can either be produced by radiation-induced polymerization and simultaneous crosslinking of hydrophilic monomers in “nanocompartments”, i.e., within the aqueous phase of inverse micelles, or by intramolecular crosslinking of suitable water-soluble polymers. The radiolytically produced oxidizing species from water, •OH radicals, can easily abstract H-atoms from the backbone of the dissolved polymers (or can add to the unsaturated bonds) leading to the formation of C-centered radicals. These C-centered free radicals can undergo two main competitive reactions; intramolecular and intermolecular crosslinking. When produced by electron beam irradiation, higher temperatures, dose rates within the pulse, and pulse repetition rates favour intramolecular crosslinking over intermolecular crosslinking, thus enabling a better control of particle size and size distribution. For other water-soluble biopolymers such as polysaccharides, proteins, DNA and RNA, the abstraction of H atoms or the addition to the unsaturation by •OH can lead to the direct scission of the backbone, double, or single strand breaks of these polymers.

## 1. Introduction

Throughout this review ionizing radiation and radiation is defined as photons or particles with sufficient energy to ionize atoms and/or molecular segments of covalent compounds. The deposition of the energy through columbic interactions (in the case of charged particles such as electron, proton, and alpha particles), and in the case of high-energy photons (through Compton scattering, photoelectric, and pair production), takes place in approximately 10^−18^ to 10^−12^ s. During this period, localized ionized and excited molecules are formed along the tracks. Most of the chemical reactions take place from 10^−12^ to 10^−1^ s. Usually, the biological processes start one second after the interaction.

There is a huge body of literature on the effects of ionizing radiation such as gamma rays from Co-60, high-energy electron from electron beam accelerators, and X-rays on the polymeric materials and composites [1,2,3,4,5,6,7,8,9,10]. For the last four to five decades, a wealth of knowledge on the effects of the radiation dose (energy per unit mass) of ionizing radiation on polymeric materials has been accumulated.

### 1.1. Fundamental of Radiation Effects on Polymers

The interactions of gamma photons, X-rays, and high energy electrons with matters induce ionizations leading to the formation of ions and expelled fast-moving electrons. While the interactions of the Co-60 gamma rays and X-rays photons through mainly photelectric, Compton scattering, and pair-production, the interactions of the high-energy electrons take place through coulombic interactions. So, despite the types of irradiation of materials with photons or with electrons, both cases produce ions and electrons. While the produced secondary and Compton, and photoelectric electrons gives rise to more ionizations, the radiolytically produced ions undergo various chemical reactions, mainly through deprotonation reactions leading to the formation of the C-centered radicals. Depending on the dose-rate, presence of oxygen, and the presence of antioxidants, these free radicals undergo various reactions. In the presence of oxygen, while the irradiation with high dose-rate such as X-rays and electron beam enhance the crosslinking reactions of these free radicals, the irradiation with low-dose, such as in the case of Co-60 gamma rays, promotes the degradation reactions through oxidations. At a low dose rate, competition reactions are established between the crosslinking reactions of these C-centered free radicals and their reactions with oxygen. The reaction of the C-centered free radicals with molecular oxygen give rise to the formation of the corresponding peroxyl radicals. Finally, these peroxyl radicals undergo various reactions leading to the degradation of the polymers.

### 1.2. The Complexity of the Chemical Structures of New Polymeric Materials Used in Advanced Technology

In present times, many new polymers have been used in advanced technology. The need to investigate the effects of the radiation dose on them is vital. These polymers and copolymers contain different molecular structures and morphologies and hence the radiation dose has different effects on them. It has been known that the presence of quaternary carbon atoms, halogen atoms (in the halogenated polymers), and C–O–C bonds on the backbone of the polymer chains, functional groups, degree of crystallinity, and the presence of the oxygen, fillers, and antioxidants, have crucial effects on the radiation chemistry of the irradiated polymers. While the absence of quaternary carbon atoms enhances the crosslinking reactions such as in the case of polyethylene, their presence promotes the scission along the backbone of the polymer chains leading to degradation. Also, the presence of C–O–C bonds like in the case of cellulosic polymers materials, the radiation induces degradation because of the scission of the O–O bonds and the production of the alkoxyl radicals. In addition to the chemical structures, the ratio of crystallinity/amorphous also plays important roles in the radiation effects of polymers. Remember that most of the radiation-induced reactions, such as crosslinking and degradation, take place in the amorphous region of the polymers. However, H-hopping on the backbone of the chain in the crystallinity region can occur. This allows for the gradual diffusion of the free radicals towards amorphous zones where they can undergo bimolecular reactions.

## 2. Fundamental and Technological Aspects of Radiation-Induced Polymerization

Polymer synthesis is the art to produce macromolecules with specific features in terms of repeat units, degree of polymerization, microstructure and topology, as well as a means to fabricate small objects (nano- or micro- particles) and macroscopic three-dimensional networks. This can be achieved either by a chain addition process or by step-growth polymerization [11]. Though most polymerization reactions proceed by chemical reactions based on conventional initiation or catalysis combined with thermal activation, alternative methods based on enzymatic or radiation-assisted processes exhibit specific features that have been explored early in the history of synthetic polymers.

Ionizing radiation is a form of energy carried by high energy electromagnetic waves (gamma or X-ray photons) by particles accelerated in an electric field (electrons, light ions, swift heavy ions (SHI)) or by elementary particles emitted from unstable atomic nuclei (α or β^−^ particles) [12]. Energy deposition occurs upon interaction with matter through a variety of extremely fast physical processes (time span up to a few fs) that end up with a physio-chemical stage during which short-lived excited species and chemical entities are generated, through a cascade process that is initially controlled by nonhomogeneous kinetics. Then, the medium responds more homogeneously in the chemical stage with solvated electrons and free radicals that exhibit longer lifetimes [13]. In-situ formation of such reactive species can be utilized to initiate chain polymerization. The subsequent stages of the chain process are, in principle, not directly affected by the ongoing radiolysis of the medium. Polymerization essentially proceeds by a chain mechanism with free radical active centers, and in some specific cases by ionic active centers generated at the end of the cascade processes of physical and chemical stages following the interaction of high energy particles and photons with molecular substrates [14].

The early articles reporting on the discovery of radiation-initiated chain polymerization of simple monomers date back to the year 1940 [7,15,16]. These studies were primarily conducted on styrene, various vinyl derivatives, and some dienes to examine the effects of the monomer structure, the nature of the solvent, and of irradiation conditions in terms of dose rate and of absorbed radiation dose. The occurrence of ionic mechanisms was also evidenced during the late 1950s for particular monomers, reaction media, and experimental conditions that could meet the demanding criteria of such reactions being very sensitive to the presence of moisture and other impurities.

Though step-growth polymerization can be efficiently triggered by the generation of acidic or basic catalysts via radiation-mediated processes, this possibility has not been studied extensively. The few known examples are essentially based on UV-visible activation of photo-base and photo-acid generators [17]. A case is found with thiol-ene polymerization that can be induced by electron beam irradiation and also enters in this class of step-wise build-up of macromolecular materials [18,19].

Currently, radiation-induced chain polymerization continues to develop as an attractive alternative to photo-initiated polymerization and UV-curing [20] in the perspective of advanced applications in material science and technology. It has been used since the early 1980’s, on a continuously increasing industrial scale, for curing thin layers of inks and coatings deposited on solid substrates (paper, plastic films, wood panels, and metal foils) using low-energy electron beams. The accelerators typically operate at a voltage between 80 and 500 kV with a strong beam current permitting short curing times and high productivity on continuous industrial lines.

The increase in the scope of applications as well as the advent of more advanced analytical methods and of dedicated irradiation equipment has fostered growing interest on a variety of new scientific issues and technological perspectives. The status on the most significant results will be discussed in this part of the review dedicated to polymerization. Another section is dedicated to radiation-induced graft polymerization as a subtopic this review.

### 2.1. Specificities of Radiation-Initiated Polymerization

Radiation-initiated processes exhibit very unique features that can be exploited for the design of basic investigations or for technological purposes:

#### 2.1.1. Instantaneous Impact of Radiation Treatment

As radiation penetrates the substrate, almost instantaneously, the beam induces the desired chemical processes. Thermal activation of chemical reactions is limited by heat transfer kinetics, and the use of catalysts is dependent on mixing conditions. Continuous, fractionated, intermittent, or pulsed irradiation can be applied to the substrate at a desired instant for inducing chemical reactions with a high degree of temporal control.

This feature is exemplified by pulse radiolysis experiments using a high-energy radiation source which allows to identify by time-resolved spectroscopic methods the nature and the concentration of various transient species such as radical cations, anions, and radicals derived from monomers and solvents, and to assess the kinetic parameters of their decay on timescales ranging from picoseconds to seconds [21,22]. Using these techniques, considerable progress has been achieved in the understanding of initiation mechanisms and in the technological use of pulse irradiation for the control of material properties [23,24].

In contrast, in the absence of any radiation stimulus, ink, paint, and resin formulations including large amounts of very reactive monomers exhibit particularly long shelf-stability, which provides a major technological advantage for the design of cure-on-command processes [25].

#### 2.1.2. Spatial Control of Radiation-Induced Effects

Beam directing devices and masking systems allow for a spatial control of the response induced within a substrate. 1D to 3D patterning can be achieved by localized polymerization at different dimension scales. The resolution depends on physical factors associated with the precision of the beam pathway and penetration, on the scattering effects of the cascading energy transfer processes, and from the diffusion of the active chemical species that participate in the reaction. Three recent works that tackle the challenges of nanometric resolution in radiation-polymerizable films can illustrate this [26,27,28].

Cationic polymerization of the solid epoxy resin SU-8 has been successfully used to pattern organic surfaces by electron beam lithography. Process parameters can be tuned to optimize the fabrication process, in terms of spatial resolution and aspect ratio for the obtained pattern. The developed technique allows for the fabrication of high-aspect ratio, surface bound nanostructures with heights ranging from 100 to 4000 nm and with in-plane resolution below 100 nm. Direct writing on glass plates as a transparent glass substrate is especially convenient for studying cell structures [29].

Irradiation of thin films of 4-vinyltriphenylamine with high-energy multiply charged Ag or Os cations induces the solid-state polymerization and crosslinking of this monomer along the ion trajectories, resulting in the formation of insoluble uniform nanowires with precise diameters. Polymerization of this monomer in the track of the swift heavy ions proceeds quite efficiently in comparison with crosslinking reaction of polymer layer treated under the same conditions. Nanowires with a cross-section lower than 10 nm were obtained and characterized after dissolution by chromatographic and spectroscopic methods to gain information on the free radical chemistry that takes place during the process [26,27].

#### 2.1.3. Random Energy Deposition

Energy deposition occurs at random as a function of the electron density in the atoms within the irradiated substrate. Early physical events are therefore nonselective with respect to the specific structure of the molecular assemblies interacting with radiation. This leads to low degrees of conversion, the validity of the assumption that the stages of chain polymerization process that follow initiation are not directly affected by the ongoing radiolysis of the medium. Larger contents in branched polymers can be expected at high conversion, as a consequence of the radiolysis of the produced macromolecules and subsequent initiation of a new kinetic chain starting from an activated repeat unit. Another consequence is the possibility to induce graft polymerization from a molecular substrate in contact with the monomer within the irradiated medium. This point is treated separately by another part of this review [21].

The nonselective energy deposition process of ionizing radiation that penetrates deeply into the reactive material is a significant advantage by comparison with the photochemical activation by UV-visible sources of pigmented formulations and composites including fillers or fibers. This is demonstrated by the capability to cure 3 cm-thick epoxy acrylate composites with about 66 wt % of carbon fiber with a 10 MeV Linac accelerator [28].

Coreaction between the polymerizable matrix and a nanoparticulate organic filler due to the nonselective activation by high energy radiation was recently evidenced for polyurethane acrylate nanocomposite materials including 1 wt % of cellulose nanocrystals which exhibit, for similar degree of conversion and glass transition temperature, a tensile strength twice as high for the electron beam cured samples compared to the UV-cured ones [30].

#### 2.1.4. Decoupling of Primary Initiation Steps from Thermal Activation

Radiation processing allows decoupling initiation kinetics from the effects of temperature on the induced chemical reactions, particularly on propagation and transfer reactions for polymer synthesis. Fundamentally, thus overcoming a critical constraint in polymerizations involving thermally activated initiators. Radiation-induced initiation, unlike chemical methods, generally generates reactive centers at a constant rate. This makes it possible to follow polymerization kinetics under stationary and various experimental conditions. Favorable conditions can thereby be achieved to ensure polymer chain propagation with minimum transfer or elimination reactions, thus reducing the risks of chain branching.

This approach has been exploited to synthesize poly(vinyl iodide), a difficult case in the class of simple halogenated polymers. Unlike other vinyl halides, vinyl iodide is quite unstable and undergoes decomposition by the action of light and oxygen producing free iodine which acts as an inhibitor. Poly(vinyl iodide) was prepared by radiation-initiated treatment of the monomer in various chlorinated solvents and isolated as a white solid with an unprecedented degree of purity [31,32,33].

Another example of the benefits arising from the use of radiation-induced initiation as an additional and versatile lever in polymer synthesis is found with butadiene and substituted analogues. These can be polymerized by various mechanisms, cationic or free-radical, in bulk state, solution, or aqueous emulsion with different kinetics and regioselectivities for monomer insertion, therefore with potential control on the microstructure [34,35,36].

As initiating species are generated randomly and softly in the solid state, in particular with X-rays and γ-radiation, it is relatively easy to polymerize conventional monomers in a variety of inclusion compounds. Various substituted butadienes have been polymerized successfully as clatrates in deoxycholic acid or thiourea crystal channels, yielding highly stereoregular poly(2,3-dimethyl-1,3-butadiene) with 97% of 1,4-trans diene units and melting temperatures as high as 272 °C [37,38,39,40]. Stereoselective polymerization of acrylonitrile also takes place in the canals of crystallized urea with formation of isotactic poly(acrylonitrile) with controlled molecular weight [41,42].

### 2.2. Basic Aspects

Many basic aspects of radiation-initiated polymerization were established during the pioneering period of this new domain of radiation chemistry by Williams, Hayashi, Okamura, Metz, Chapiro, Machi, Stannett, and Charlesby, to name some of the most significant contributors [7,16,43,44]. Most conventional monomers have been studied both as bulk substrates in the liquid or in the solid state, as well as in solution or in heterophase systems such as aqueous suspensions or emulsions.

In dilute monomer solution or in liquid heterogeneous systems, radiolysis of the solvent generally drives the initiation process, whereas bulk monomers produce ionized species, i.e., radical cations, and electronically excited species which produce in turn free radicals, by neutralization and by monomolecular dissociation, directly or after charge recombination.

**Scheme 1:**
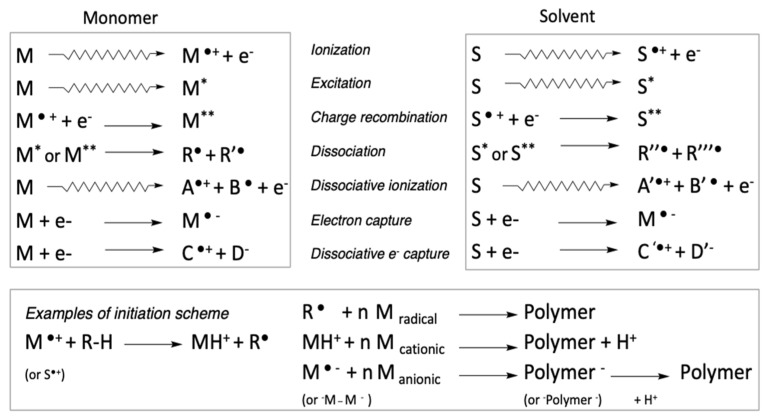
Simplified description of radiation-initiated polymerization for monomer M in the bulk or dissolved in solvent S. Depending on the chemical nature of the monomer, the purity of the medium and on the reaction conditions, chain propagation is initiated by appropriate free radical or ionic active centers.

A simplified picture of the main stages of the complex mechanism leading to the initiating species is presented in Scheme 1.

The fast electrons produced during the primary ionization of the substrate gradually lose their energy during secondary acts thus causing the formation of numerous ions and electrons of high energy. This primary electron e^-^ is transformed into a thermalized electron e^−^_th_, i.e., in thermal equilibrium with the medium. The thermal electron can be trapped by radical cations formed from monomers M^●+^ or from the solvent S^●+^ that are converted into electronically excited molecules with an energy excess of 8–5 eV higher than the strength of covalent bonds in organic molecules (~3 eV). The molecule in a dissociative excited state therefore decomposes into free radicals, some of which are able to initiate the propagation. Energy deposition in some cases is not sufficient to induce ionization, the resulting excited molecules M** can dissociate into free-radicals. The formation of M* and M** are estimated to occur with similar probabilities [7].

Those free radicals will be subject to diffusion-controlled recombination, soon establishing a steady state concentration in the chemically homogeneous system. The specific case of acrylate monomers that involve the reduction of the conjugated unsaturation by thermalized electrons will be discussed in the forthcoming section. The rate for the generation of radical cations by ionizing radiation is considered to be two orders of magnitude lower than that of free-radical formation, but the recombination constants for ions (ion and counter-ion) are approximately two orders of magnitude higher than those for free radicals. The stationary concentration of ions can therefore be estimated to be about 100 times lower than that of free radicals [7]. Consequently, radiation polymerization proceeds mainly by a free-radical mechanism unless specific conditions are met.

Introducing onium salts such as triaryl sulfonium or diaryl-iodonium associated with low nucleophilicity counter-anion in the reaction medium allows generating strong acids and/or carbenium that can initiate cationic processes more efficiently [45,46,47].

#### 2.2.1. Free Radical Polymerization

Radiation-induced polymerization has been studied with many monomers irradiated as bulk liquids, in solution, aqueous suspension or emulsion, the gas and solid crystalline or glassy state, as for other methods of initiation (conventional, thermal, photochemical initiation, etc.), potentially with the additional advantages developed in the preceding section.

Intensive research has been conducted from 1960 to 1980 on monomer precursors of commodity polymers, such as ethylene at various pressures and temperatures, [48,49,50], in solution or in supercritical CO_2_ [51] and at pilot-scale [52,53,54,55,56]. It was confirmed that short chain branching is considerably reduced by γ-ray initiation at low temperature [57].

Styrene is a versatile monomer in terms of polymerization mechanism. Depending on temperature and the nature of the solvent, a free radical [58,59], or a cationic mechanism [60,61,62] can operate. Most convincing arguments supporting the nature of the dominant mechanism were obtained by experiments using specific inhibitors, such as benzoquinone or diphenylpicrylhydrazyl radical (DPPH) for free radicals, or water, methanol, and ammonia for cationic species. Kinetic considerations were based on the order n of the dose rate (Ḋ), dependence of the polymerization rate of the monomer, (R_p_), expressed as R_p_ = K·Ḋ^n^·C_M_ and of the dependence of measured molecular weight of the isolated polymers on Ḋ, monomer concentration C_M,_ and temperature. It was however difficult to draw clear conclusions from several studies, since both mechanisms can take place simultaneously, with a complex dependence on reactions parameters and strong sensitivity of cationic entities to trace amounts of impurities present in the reaction medium. Copolymerization experiments with monomers compatible of only one of the two possible mechanisms were used to obtain additional evidence on the nature of the effective propagating centers [63].

**Figure 1:**
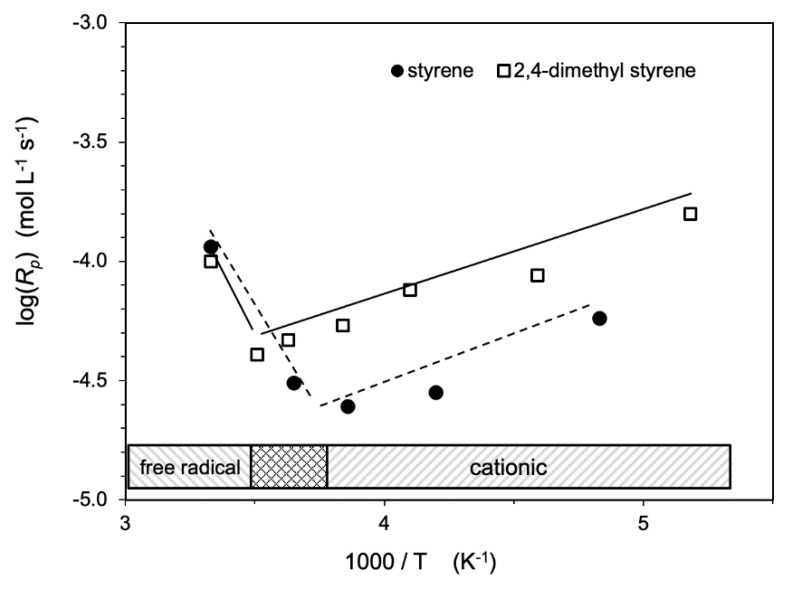
Temperature-dependence of the radiation induced polymerization of styrene and 2,4-dimethylstyrene revealing the temperature ranges favoring the occurrence of a cationic or of free radical mechanism (after [64]).

A detailed study on the temperature-dependence of the polymerization rates for styrene and 2,4-dimethylstyrene upon irradiation in chlorinated solvents showed that an ionic mechanism is involved in the temperature range in which the activation energy is negative (Figure 1) [64].

Advanced studies on the γ-ray induced polymerization of styrene under pressure were conducted from 1970 to 1983 to clarify mechanistic aspects and to determine kinetic constants and thermodynamic parameters such as the activation volume, as a function of temperature and pressure in the bulk state and in emulsion [65].

Rubbers based on butadiene were also studied quite intensively from the late 1950s to the early 1990s in solution and in emulsions [35,66,67,68,69,70,71,72].

Access to specialty polymers was explored with vinyl acetate and butyl acrylate polymerized in emulsion [70,73,74], or butyl acrylate copolymerized with butadiene, or polymerized at the surface of rubber particles to improve their compatibility with host matrices [75]. Water soluble poly(acrylamide) was obtained by γ-ray induced polymerization in various hydro-organic solutions to improve its efficiency as a flocculant [76].

The unique properties of fluorinated polymers stimulated studies on new synthetic approaches based on radiation-induced homo- and copolymerization of tetrafluoroethylene [77,78,79], hexafluoropropene [80], methyl trifluoroacrylate [81,82], in bulk conditions or in emulsion have been studied.

Various other studies devoted to monomers including heteroatoms, such as diphenylvinyl phosphine oxide obtaining polymers with polar yet aprotic properties useful as a host matrix [83], vinylsulfonamide to produce functional polymers useful as synthetic fibers, adhesives, ion exchange resins [84], vinylbenzyltrimethyl ammonium chloride, as a precursor of chemically stable separation membranes and resins [85], and highly reactive multifunctional acrylates including Si, Sn, or Ge atoms as a consequence of their high stopping power and/or for potential applications as high refractive index materials [86,87,88] have been reported.

Several new polymers or polymers with a specific microstructure have been synthesized by radiation-induced polymerization. Many of those products exhibit excellent properties with potential practical uses, however little if any commercial products are currently on the market.

Radiation-induced polymerization also proceeds in bulk solid state in spite of the limited molecular diffusion for the propagation steps and of an expectable poor energetic efficiency. Topochemical effects and confinement in solid guest materials have stimulated various fundamental investigations. Contrasting behaviors were observed depending on monomer orientation and on the gap between the reactive functions within the crystal lattice. In some systems, polymerization seems to be initiated within crystalline defects, and continues as the formation of amorphous polymer domains affects the original crystalline lattice. In some favorable systems, the relative orientation of monomers prefigures the order of repeating units of the resultant polymer [44].

The potentialities of these self-orienting polymerizations has stimulated more studies on MMA [89], a long series of articles by Hardy (e.g., *N*-vinyl succinimide in liquid and solid state [90,91]), maleimides [92], vinyl chloride and trichlorofluoroethylene [93,94], methacrylamide, allylurea and *N*-vinylpyrrolidone [95], diacetylenes [96,97], and various monomers confined in clathrates, as already mentioned in the preceding section.

#### 2.2.2. Ionic Polymerization

Due to the formation of active species of different natures, free radical and ions, the actual and precise propagation mechanism requires careful investigation. Ionic polymerizations are sensitive to the presence of impurities that may arise and to temperature, which activates the dissociation of propagating species but also favors transfer reaction. 

#### 2.2.3. Cationic Polymerization

The radiation-induced polymerization of styrene is a good example of the intense debates which took place when comparing the results obtained by different teams from the radiation chemistry community [59]. The kinetic data obtained with styrene thoroughly dried by distillation over Na-K alloy were quite different from the values reported from previous studies in conditions where the radical mechanism was predominantly operating. In dry medium the free radical mechanism can be ruled out on the basis of the following arguments. Addition of small amounts of water and ammonia caused a remarkable reduction in the rate of polymerization (R_p_), whereas the activation energy for propagation was nearly zero between −20 and +80 °C and R_p_ was proportional to (dose rate)*^n^*, with 0.8 < *n* < l, while the molecular weight was independent of dose rate. These results strongly support the intermediacy of ionic species. The cationic or anionic nature of the mechanism was further clarified by performing copolymerization of styrene with α-methyl styrene and with isobutyl vinyl ether which proved to be effective, hence confirming the occurrence of a cationic mechanism. This was established by additional evidence based on the retardation propagation by the addition of ammonia and diethyl ether in the medium. Radical scavengers such as DPPH and oxygen also retarded the polymerization, suggesting the intermediacy of an ion-radical in the initiation stages. It can be concluded that the propagation mechanism involves free ions.

Many other advanced studies conducted on vinyl and diene derivatives irradiated in the liquid or in the solid states include ethylene [97], isobutene [60], butadiene [34,98], α-methyl styrene, pinene and other terpenes, vinyl ethers [99,100]. Cyclic oligo-acetals [101] and cyclic ethers such as trio-, tetra-, and penta-oxane [102,103] were studied during the same period.

The radiation-initiated ring-opening polymerization of oligocyclosiloxanes has also been considered to occur via a cationic mechanism on the basis of the molecular weight distributions that show limited back-biting reactions. The intermediacy of a chelated silicenium has been proposed to account for the small differences in reactivity observed in the reaction rates of the cyclic oligomers and distribution of reaction products, in contrast with chemically initiated polymerization [104].

Probably, the more important results from the viewpoint of the potential applications is the cationic polymerization of epoxides [105,106] which will be further discussed later in this review.

#### 2.2.4. Anionic Polymerization

Nitroethylene is among the most representative monomer proved to polymerize by radiation-initiated anionic process. Its reactivity has been studied during the period 1966–1969 [107,108]. The anionic mechanism involves free ions. Its polymerization kinetics were investigated by using hydrogen bromide as an anionic scavenger. G_(initiation)_ was about 0.3 µmol J^−1^, which is much larger than the value (0.01 µmol J^−1^) obtained for many ionic polymerizations of unsaturated hydrocarbons. This difference may be explained by the large dielectric constant of the medium and high electron affinity of this particular monomer. Additional information on the mechanism and on the lifetime of transient species was obtained with pulse radiolysis experiments conducted at low temperature [109]. β-nitrostyrene [110] has also been confirmed to polymerize by an anionic mechanism.

#### 2.2.5. Controlled Free Radical Polymerization

Since the early developments of controlled free-radical polymerization, radiation-mediated methods have been tested and developed in tandem with RAFT because of advantages of the soft, regular and penetrating mean of activation provided by γ irradiation [111]. This combined technique is particularly useful to conduct mechanistic and kinetic investigations [112,113]. From a preparative standpoint, it has been demonstrated as a unique approach to synthesize some polymers with the features of controlled polymerization [114]. The potentialities for the precise modification of various types of surfaces and substrates by radiation grafting are demonstrated by an increasing number of reports [115,116].

In conclusion to this section, the potential advantages of radiation-induced polymerization as an industrial method for the synthesis of commodity or low value-added polymers has not been confirmed to a sufficient level nor with significant economic benefit to allow industrial development for the production of commodity polymers. One of the basic limitations of the radiation-induced process is that the advantages in terms of structural features of polymers in specific conditions are lost at higher conversion, as a consequence of radiolytic effects on the polymer formed in situ.

However, the research that was conducted during these years has produced quite important results with significant impact in polymer science. The radiation-induced grafting and radiation curing by cross-linking polymerization, negative-tone lithography as well as specific applications based on radiation-induced polymerization in confined or multiphase components and media have been established as valuable technological options for specific and value-added applications.

### 2.3. Radiation-Induced Cross-Linking Polymerization

**Scheme 2:**
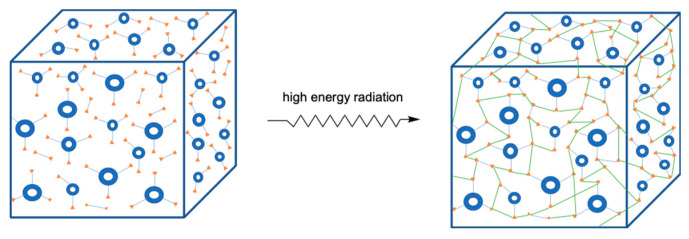
Radiation-induced cross-linking polymerization of blends based on reactive prepolymers and multifunctional monomers.

The curing of mixed monomers and prepolymers formulations is by far the largest application domain of radiation-induced polymerization. Cross-linking polymerization is initiated almost instantaneously upon interaction with the triggering beam to form covalent polymer networks resembling those in thermosets (Scheme 2).

Solvent-free formulations of adhesives, inks, overprint varnishes, coatings, and paints can be cured by UV- or radiation-induced crosslinking polymerization. These methods are gaining shares over conventional solvent-based and/or heat-curing processes which are gradually phased-out in graphic arts and coatings industries because of their reduced environmental footprint and better sanitary profile (reduced energy consumption and volatile organic compounds emission) [117].

Compared to UV-visible photon sources and accelerated electrons, high energy X-rays and γ radiation indeed penetrate more deeply in heavily pigmented inks and paints and in composites with high levels of fillers or fibers. High energy radiation can cure sealants which are sandwiched between non-transparent materials. In addition to this advantage relating to the physical characteristics of ionizing radiation, as no primary initiator nor photosensitizer are needed to generate free radicals upon irradiation, the risk of producing toxic extractable chemicals is considerably reduced. The resulting formulations and the cured materials are consequently even safer for food packaging, biomaterials, and environmentally friendly applications [118].

These specific features are particularly well-exploited in the remarkable restauration process for damaged archaeological objects made of wood, and for some other types of weak artistic pieces with a porous structure. After infusion of a restorative monomer-based resins, the unique in-depth chemical effects of high energy radiation result in the consolidation of the artefacts by solidification of the liquid by polymerization, all stages of the process being conducted under mild conditions [119].

#### 2.3.1. General Description

Two main classes of monomers are commercially available depending on the type of polymerization mechanism. The first group is comprised of monomers fitted with ethylenic unsaturations, mainly acrylates and methacrylates, but also styrene and its derivatives as well as some N-vinyl lactams. Most of them undergo fast free radical polymerization. The second group includes molecules bearing epoxy or vinylether functionalities for polymerization mechanisms mediated by cationic centers. The architecture, the chemical nature of the backbone, and number of monomer units attached to these monomers and prepolymers have a strong impact on their reactivity and on the physical properties of the cured material. The requirements in terms of tensile strength, flexibility and elongation, gloss, scratch and solvent resistance, adhesion to substrate or the reinforcing materials durability upon photochemical and hygrothermal ageing may differ considerably, depending on the domain of application.

**Scheme 3:**
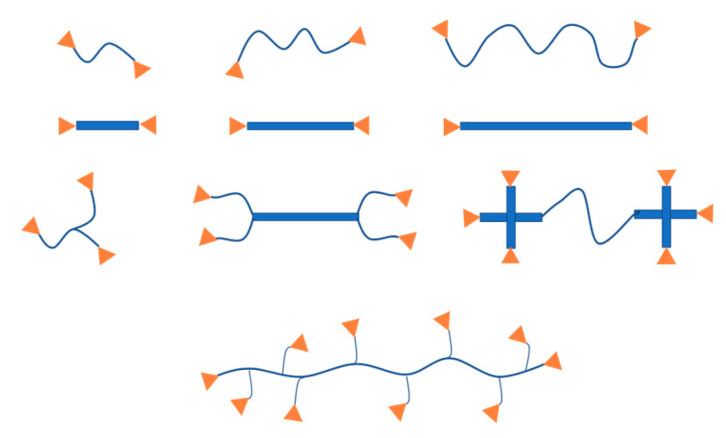
Generic structures of monomers and prepolymers with soft or rigid segments used in radiation-curable blends.

The different generic structures represented in Scheme 3 exist with aliphatic or aromatic hydrocarbon scaffolds, with linear or branched polyethers, polyesters, polyurethanes, silicones, with various molecular weight and number of attached monomer units. The balance between the rigidity of the scaffold’s segments and the final crosslink density strongly influences the thermo-mechanical properties of the obtained networks. Mono- or multifunctional reactive diluents which are primarily added to the prepolymer blends to adjust its rheological properties also influence the curing reactivity by acting on the initial concentration in monomer groups and on the density of cross-links. A variety of reactive additives specifically developed for radiation-curing applications help to improve surface properties.

#### 2.3.2. Initiation Mechanisms

**Scheme 4:**
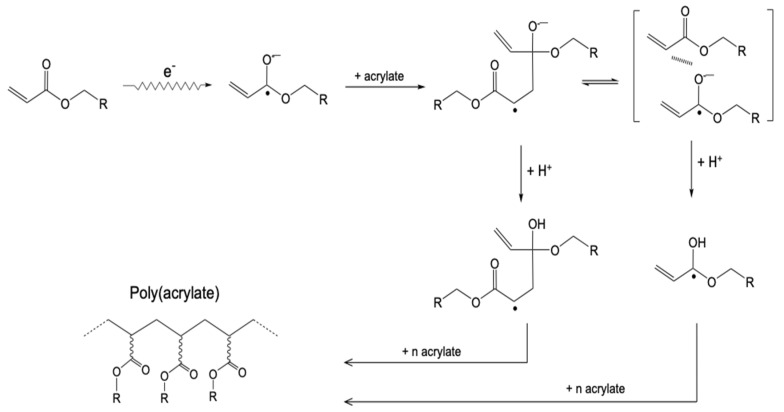
Proposed mechanism for the initiation of acrylate polymerization upon exposure to high energy radiation [122].

Pulse-radiolysis experiments performed with simple acrylate monomers, either in the bulk state or in solution in various solvents provide a better understanding of the pathways leading to effective initiation of polymerization. Irradiation of acrylates and methacrylates were studied in cyclohexane solution [21,120,121]. Diacrylates were studied in n-butyl chloride solution [122] and water-soluble diacrylates in diluted aqueous solution [123,124]. Kinetic and mechanistic information are obtained by analyzing the decay of the transient species known to appear upon irradiation of the solvent. The bulk monomer generally behaves in a more complex manner, since it is composed of various structural moieties. A schematic depiction of the mechanism operating aliphatic acrylates such as tripropyleneglycol diacrylate irradiated in the bulk state can however be proposed by the sequence of events shown in Scheme 4. Attachment of a thermalized electron to the carbonyl of acrylate esters, and the subsequent formation of a radical anion dimer either represented by a charge transfer complex, or by a covalent adduct. Protonation of the corresponding radical-anions leads to the effective neutral free radical species that initiate propagation. The direct formation of free radicals by homolytic dissociation of electronically excited monomer moieties is considered to be a minor initiation pathway. 

**Scheme 5:**
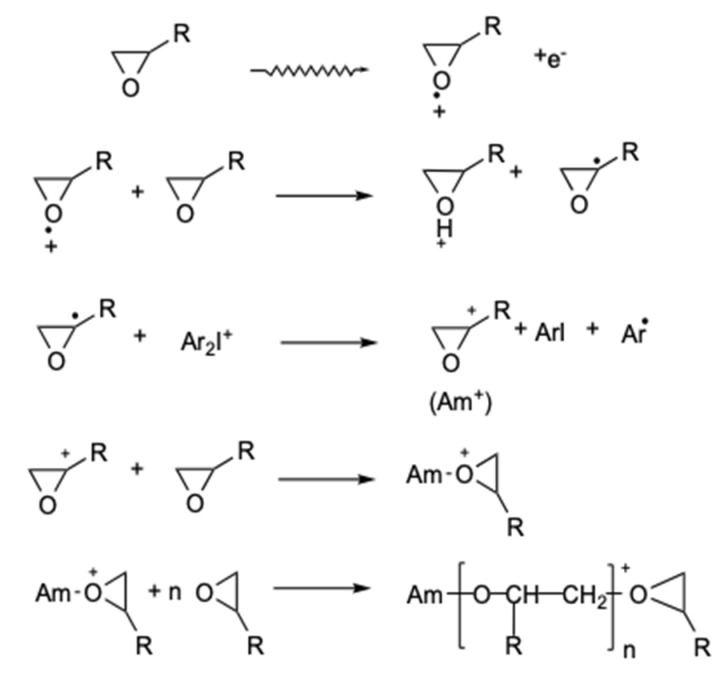
Simplified mechanism for the formation of the initiating species for epoxy monomers in presence of diaryliodonium salt upon exposure to high energy radiation (counter-anions of the onium salt are omitted in this scheme for the sake of simplicity).

As mentioned earlier, radiation-induced cationic reactions are sensitive to the chemical nature of the medium and to the presence of impurities. Cationic polymerization is preferably conducted in the presence of onium salts to ensure efficient initiation. Many of the diaryliodonium and triaryl sulfonium salts which are used as oxidative coinitiators in the radiolytic process can also operate by direct and selective UV photolysis [125]. The two activation processes are actually quite different. The dominant pathway in an irradiated medium rich in monomers and where energy deposition occurs at random, is the production of free radicals which can be oxidized by electron transfer to the onium salt, generating carbenium cations (Scheme 5) [126]. Reduction of the onium salt can also occur from its interaction with solvated electrons (direct reduction pathway). More complex radiolytic processes mechanism were confirmed by pulse radiolysis experiments on phenyl glycidyl ether in the presence of an iodonium salt [126]. The decomposition of the low nucleophilicity counter-anions, such as hexafluorophosphate or hexafluoroantimonate, has been also been reported based on early observations of these systems, but still remains to be elucidated [127,128].

At the dose rate provided by high energy radiation sources, preferably electron accelerators, the number of active centers per time unit is generally sufficient to ensure the fast and extensive polymerization of the organic binder. In this situation, inhibition by monomer stabilizers, by atmospheric oxygen in the case of free radical initiation, or by nucleophiles and moisture that deactivate cationic centers, are overcome during the first instants of irradiation by the formation of a larger amount of reactive species. Thermal aspects related to the exothermal flux induced by the polymerization and by the enthalpic conversion of the absorbed radiation are crucial since translational and segmental mobility is needed to ensure the development of covalent networks by cross-linking polymerization [129]. A precise control of temperature profiles is therefore required during the elaboration of high-performance materials capable of withstanding high service temperature and exhibiting high mechanical properties.

**Scheme 6:**
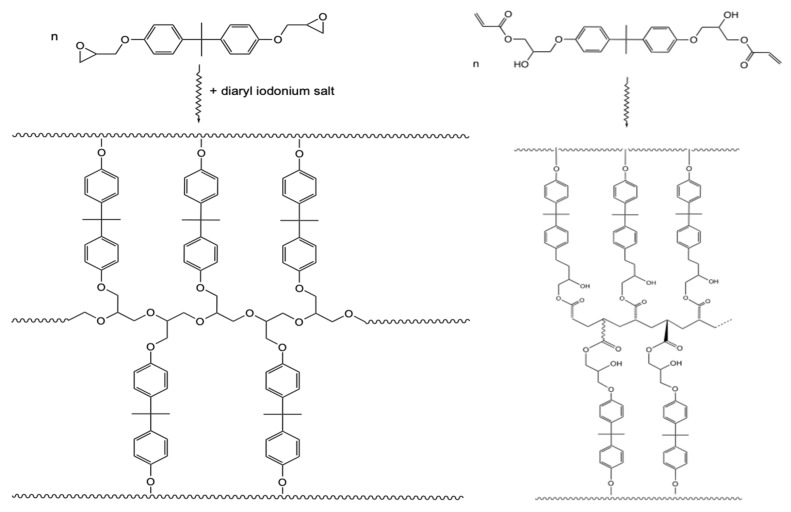
Molecular structure of bis-phenol A diglycidyl ether (DGEBA), of bis-phenol A epoxy acrylate (EPAC) and of the networks resulting from their radiation-initiated crosslinking polymerization.

The simple bisphenol-A derived diepoxy monomer DGEBA, and the diacrylate EPAC shown in Scheme 6 are representative models of the radiation-curable resins used for such applications. Many studies have examined their reactivity upon radiation-induced cationic and free radical polymerization, respectively. The idealized structure of the corresponding networks emphasizes the very high cross-linking density in the two types of resulting networks which include quite rigid bisphenol-A segments.

Beyond the already mentioned differences due to the chemical nature of the monomers and in precise initiation mechanisms, a number of other contrasting features associated with the radiation treatment or with the physical characteristics of the reactive system may exert, in a direct or indirect manner, an influence on the build-up of the network and on the final properties of resulting material.

#### 2.3.3. Gelation and Vitrification during Network Formation

The combination of spectroscopic and thermophysical analyses allows for a more precise description of the curing behavior. The following results highlight the key aspects of polymerization kinetics and network formation.

**Figure 2:**
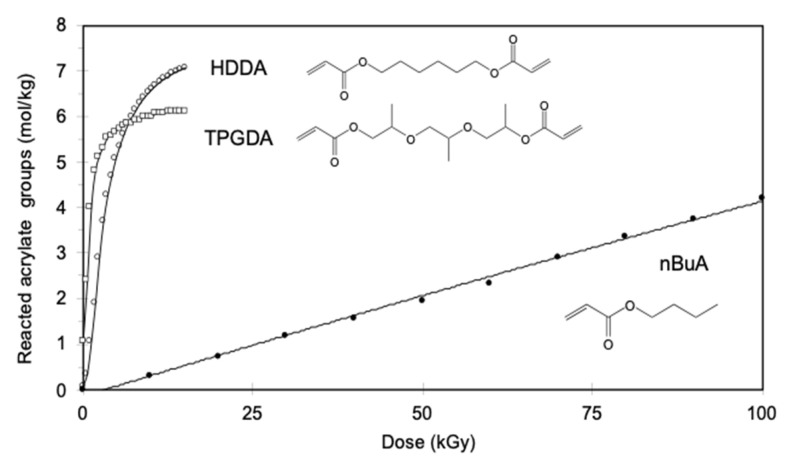
Kinetic profiles of acrylate consumption in monomer films as a function of EB radiation dose (nBuA (●), tripropyleneglycol diacrylate (TPGDA) (☐) and hexanediol diacrylate (HDDA) (○).

Crosslinking-polymerization of blends based on multifunctional monomers and prepolymers undergo macroscopic gelation at rather low conversion degree [130]. The resulting auto-acceleration or Trommsdorff effect is exemplified by comparing the conversion plots for a monoacrylate to those recorded of diacrylates. Butyl acrylate (*n*BuA), hexanediol diacrylate (HDDA) and tripropyleneglycol diacrylate (TPGDA) have comparable acrylate functionality contents in the bulk state (between 6.7 and 8.8 mol kg^−1^). The conversion plots of Figure 2 were obtained by FTIR monitoring after cumulative application of 10 kGy e-beam dose increments at the same dose rate of 11 kGy s^−1^. The profiles show that the initial polymerization rates are 30 times faster for HDDA, and 60 times faster for TPGDA than that of nBuA. Since the viscosities of the bulk monomers are not very different, assuming that the generation of initiating species and that the intrinsic reactivity of the acrylate functions are similar for the three monomers, the contrasting initial polymerization rates would essentially result from differences in the steady-state concentration in free radicals due to the much slower bimolecular termination rate in multifunctional monomers.

This phenomenon explains to a large extent the extremely fast polymerization of acrylate-based ink formulations for graphic arts and for coatings for optical fibers, with curing speeds under UV or high energy radiation as high as several hundreds of meters per minute on high-performance industrial lines [131,132,133].

Dramatic changes in the rheology arise as cross-linking polymerization progresses in solvent-free radiation-polymerizable compositions. Initially, the blends viscosity ranges from 0.5 to 5 Pa s at the application temperature which facilitates the spreading of the blend onto the substrate or the impregnation of fibers or fillers during the fabrication of composite materials. Network formation proceeds with a gradual reduction of mobility from the fluid state, to a gel, and eventually to a vitreous material. The viscosity increases by several orders of magnitude until solidification, at first with the positive influence on polymerization kinetics discussed in the previous section, and then by a strong reduction of polymerization rate as the monomer is depleted and as the material approaches vitrification.

Comparison of the EB-curing kinetics for an aliphatic polyurethane triacrylate (APU) having an initial acrylate content of 3.5 mol kg^−1^ with an aromatic epoxy-diacrylate (EPAC) with a higher initial acrylate content (about 6 mol kg^−1^) is quite instructive. These experiments were conducted under conditions minimizing the thermal effects due to polymerization exothermicity by applying small dose increments onto thin films of the prepolymer mixtures cast on NaCl windows [134].

**Figure 3:**
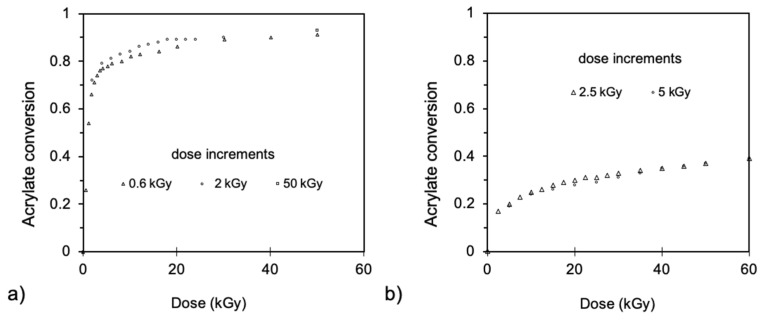
Kinetic profiles of acrylate consumption in prepolymer films as a function of EB-radiation dose: (**a**) APU and (**b**) EPAC.

While the polyurethane acrylate possesses a flexible backbone, which yields a soft material upon curing, the epoxy acrylate tends to form a glassy network even at low conversion levels. The kinetic profiles illustrate quite clearly the effects of incipient vitrification that occurs at different conversion levels. The acrylate plot shows a steep increase in monomer conversion to 0.75 for a dose lower than 10 kGy (Figure 3). The curve then levels off to a plateau with a conversion value about 0.9. The plot for the aromatic epoxy diacrylate indicates that the fast-initial stage has abated at a low conversion level of 0.2, the conversion approaching 0.4 only for a dose of 60 kGy. At this stage, the concentration of unreacted acrylates is 3.6 mol kg^−1^, a value that is even higher than the acrylate concentration in the unreacted APU sample. The poor reactivity observed in spite of the large concentration of monomer reveals the influence of incipient vitrification that hinders propagation.

As curing was performed at quasi-isothermal conditions, vitrification took place in the APU material at conversion levels slightly above 0.7, the critical value at which the kinetics started to level off. The vitrification phenomenon took place at much lower conversion levels (typically 0.2) in the more rigid EPAC prepolymer when cured at room temperature.

**Figure 4:**
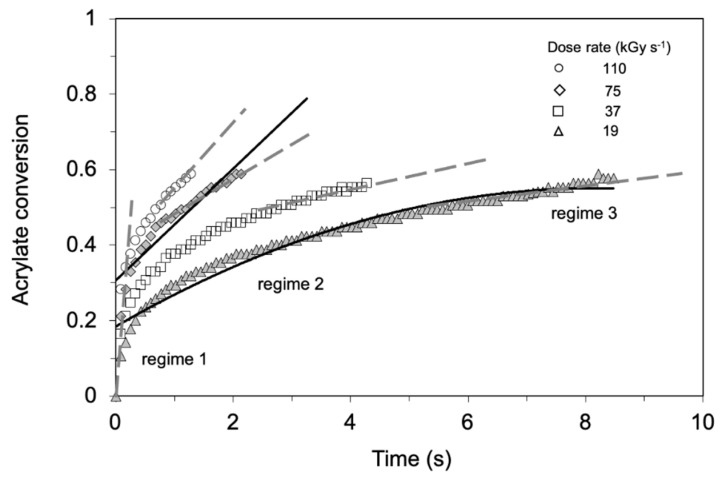
Kinetic profiles of acrylate consumption for EPAC prepolymer processed at different dose rates.

The dose rate-dependence of the quasi-isothermal kinetic profiles on polymerization kinetics of the EPAC diacrylate at EB currents corresponding to dose rates between 19 and 110 kGy s^−1^ is shown in Figure 4.

Three kinetic regimes were considered for each plot. At low conversion, the initial polymerization rate was proportional to the square root of the dose rate Ḋ, as is expected from the bimolecular termination kinetics occurring for free radical chain processes in fluid media (Equation (1)).

(1) $$R_{p}=\frac{k_{p}R_{init}}{2\sqrt{k_{t}}}\left[C=C\right]\propto \frac{k_{p}}{2\sqrt{k_{t}}}\left[C=C\right]{\dot{D}}^{0.5}G\left(R^{\cdot }\right)$$

where *k*_p_: the reaction rate constant of the propagation reaction;

*R_init_*: the rate of initiation reaction;

*k*_t_: the reaction rate constant of the termination reaction;

Ḋ: dose rate;

G(R^●^): G—value of carbon centered radicals;

*R*_p_: reaction rate of the propagation reaction;

C=C: initial concentration of vinyl group.

Deviations from this initial regime were evidenced as soon as the initial slope is affected by incipient vitrification. The curved part of the profile is assigned to a transition regime which evolves to the final segment where the polymerization rate is directly proportional to the dose rate. This corresponds to a monomolecular termination by occlusion of the growing free radicals in the vitrified matrix.

UV-curing experiments performed under iso-thermal conditions at controlled temperatures clearly establish a correlation between the curing temperature and the conversion level at the beginning of third regime. Dynamic mechanical analysis of samples prepared with this critical conversion level indeed show that the glass transition of the network does not differ from the curing temperature by more than 5 °C [135]. These results stress the importance of the relation between the effective curing temperature and the conversion dependence of the glass transition on the material network. From a practical view, it is therefore crucial to control the thermal profile in the processed materials along with the radiation treatment, if one wants to take advantage of radiation processing as an out-of-autoclave alternative to conventional curing of thermosets [136]. To achieve the desired degree of curing without external heating, there should be a finely-tuned interplay between the control of the polymerization exotherm (typically 80 and 100 kJ mol^−1^, for acrylates and epoxy functionality, respectively), the energy conversion from the deposited radiation dose, the heat and radiative exchanges with the surrounding environment, and the conversion dependence of vitrification.

**Figure 5:**
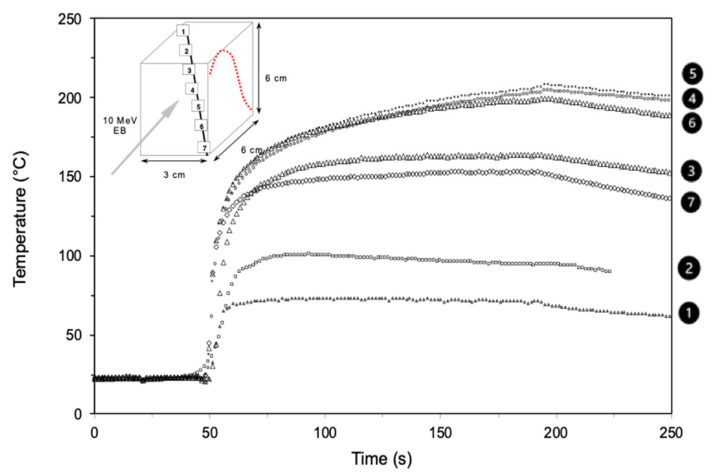
Position of thermocouples in a 125 g EPAC resin sample contained in a thin-walled aluminum box to (dotted line indicating the dose-depth deposition profile) and plots of the variations of the temperature in the sample submitted to a 50 kGy dose of 10 MeV electrons.

The large thermal effects occurring upon exposure to the electron beam of a 20 kW/10 MeV accelerator have been measured in a 125 g EPAC sample fitted with a series of thermocouples placed in thin-walled aluminum box and treated with a single 50 kGy dose in the configuration represented in Figure 5a. The plots of Figure 5b show that in central positions where energy deposition is maximal and where heat dissipation is minimal, the raise in temperature can be as high as 180 °C in the sample which was at room temperature before irradiation. The temperature increase within the sample increases is essentially due to the polymerization reaction. Assuming that the heat capacity of the epoxy resin is about 2 J K^−1^ g^−1^ [137], the increase due to the absorption of the 50 kGy dose would amount to about 25 °C at best, a value calculated for a strictly adiabatic process which is not the case in practice.

**Figure 6:**
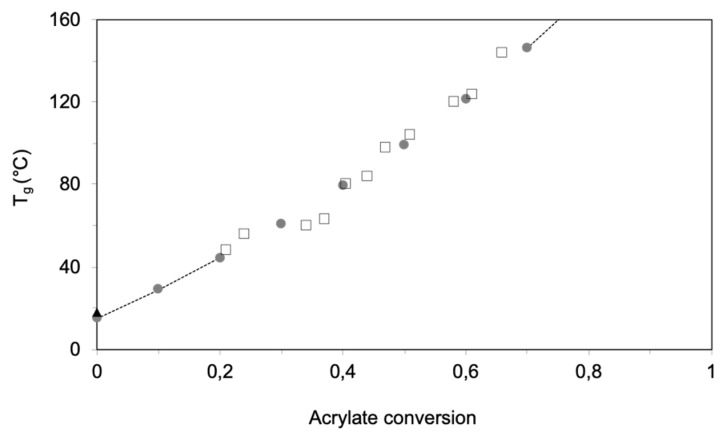
Plot of the *T*_g_ (tanδ maximum in DMA spectrogram) as a function of acrylate conversion for EB-cured EPAC materials (for various EB doses, dose rates, and dose increments). The dotted line corresponds the simulation based on DiBenedetto’s model.

This explains why the glass transition temperature (*T*_g_) of EB-cured EPAC networks can reach values as high as 180 °C. The conversion-dependence of *T*_g_ determined by Dynamic Mechanical Analysis of thin films and bar-shaped specimens treated under various irradiation conditions exhibits a monotonous increase suggesting a continuous build-up of the network, as a consequence of the increase in crosslinks density (Figure 6).

The variations can be satisfactorily described by the DiBenedetto or Pascault–Williams relation (Equation (2)), where *T*_g_ is the glass transition temperature of network at conversion degree x, *T*_g0_ is the glass transition temperature of the uncured resin (*x* = 0), *T*_g ∞_ is the glass transition temperature of the fully reacted resin (*x* = 1), and λ is a structure-dependent parameter with value between 0 and 1), as represented by the continuous line in Figure 6.

(2) $$\frac{T_{g}-T_{g0}}{T_{g\infty }-T_{g0}}=\frac{\lambda x}{1-\left(1-\lambda \right)x}$$

The established relation between *T*_g_ vs. monomer conversion can be used to describe the variations of viscosity in the sample subject to curing by using the Williams–Landel–Ferry (WLF) model expressed by Equation (3),

(3) $$\frac{\eta_{T}}{\eta_{T_{g}}}=exp\left[\frac{C_{1}\left(T-T_{g}\right)}{C_{2}+\left(T-T_{g}\right)}\right]$$

The WLF relation is commonly used to describe the increase in viscosity η_T_ when the temperature of a softened polymer approaches *T*_g_ from higher temperatures *T*, η_Tg_*C*_1_ (unitless) and *C*_2_ (°C) being numerical parameters.

By adapting the reading of this description to a curing process, at a given polymerization temperature *T*, it is possible to relate the decrease in segment mobility due to the progress of conversion vitrification that gradually shifts the network *T*_g_ to higher temperatures.

A model was developed for predicting the *T*_g_ of a volume element in a radiation-cured material. Studies on temperature effects on isothermal polymerization kinetics for EPAC monomers showed that only regime 1 was thermally activated. Once vitrification has occurred in the sample, propagation appeared unsensitive to thermal effects. The kinetics of isothermal polymerization is modeled on the basis of the two extreme kinetic regimes observed in the conversion vs. dose plots and from the assumption that a linear combination of the 2 regimes can describe satisfactorily the transition regime, as written in Equation (4) [138],

(4) $$R_{p}={\left[M\right]}_{0}\left(1-\pi \right)\left\{\alpha \;{\left[A\;{\dot{D}}^{0.5}e^{-\frac{E_{a}^{1}}{RT}}\right]}_{}+\left(1-\alpha \right)\left[B\dot{D}\right]\right\}$$

the dependence of the weighing factors α and (1 − α) for each extreme regime as a function of the progressive change of the *T*_g_ being expressed through the WLF equation by means of Equation (5), where *f*_N_ is a normalization factor.

(5) $$\alpha =f_{N}exp\left[\frac{C_{1}\left(T-T_{g}\left(x\right)\right)}{C_{2}+\left(T-T_{g}\left(x\right)\right)}\;\right]$$

The crosslinking polymerization of multifunctional monomers is known to yield brittle matrices, therefore limiting the development of this technique for the production of high-performance composite materials. Among the various possible causes of the brittleness, the spontaneous formation of nanoheterogeneities during radiation-initiated polymerization. Solid state ^1^H NMR relaxation experiments in radiation-cured materials prepared from model difunctional monomers allows one to distinguish two phases inside the materials: one consisting in rigid domains, and a second one with higher local mobility and distinct relaxation kinetic features [139]. The two-component decay of the transverse magnetization are associated with one short and one long *T*_2_ value which can be assigned to the highly cross-linked and the loosely cross-linked phase, respectively. The influence of acrylate conversion on the relaxation behavior of cured samples was examined to describe the gradual evolution of the different domains, in terms of local mobility and associated fraction of material, along the curing process.

**Figure 7:**
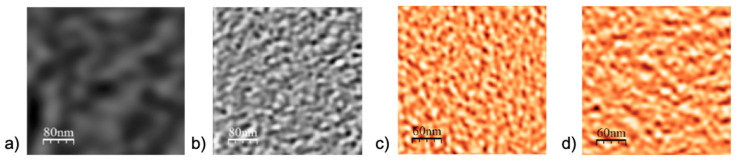
Height (**a**) and phase contrast (**b**–**d**) AFM images recorded in tapping mode of EB-cured epoxy diacrylate (EPAC) samples at conversion levels x = 0.41 (**a**,**b**), 0.46 (**c**), and 0.59 (**d**).

AFM analysis of the EPAC samples in the phase imaging mode provides a complementary picture of the network with indications on the actual dimensions of the soft and rigid domains. Topographically, the images reveal a very flat surface with a roughness of 0.2 nm, whereas the phase contrast picture highlights a more complex network structure [140]. Dense nodules appear very early at the brighter zones with a mean cross-section of about 15 nm, whereas the darker interstitial zones correspond to loosely crosslinked and swollen domains (Figure 7). Measurements of the number, Feret’s diameter, and cross-section area of the rigid domains reveal that nanogel clusters are initially embedded in a soft gel, undergoing limited evolution by growth and by aggregation up to a limiting size at higher conversion levels. Nucleation within the monomer rich domains further continues up to a 50% conversion, together with limited growth by aggregation of adjacent particles. Polymerization then continues in interstitial domains, generating a stringy network with some discrete low conversion domains.

Differential scanning calorimetry (DSC) of UV- and EB-cured diacrylate materials exhibiting a fractional degree of conversion ranging from 0.1 to 0.8 have been analyzed in the light of these results [140]. Two main second-order thermodynamic transitions were observed by using the temperature-modulated mode of DSC which avoids perturbations coming from irreversible heat exchanges, as postpolymerization enthalpy. The bimodal distribution of transition temperatures observed as fused peaks in the thermograms representing the first derivative of reversible heat capacity dC_p,rev_/dT is satisfactorily resolved by a two-component fit, allowing for a quantitative exploitation of the data in terms of Gaussian contributions, with a central relaxation temperature and a peak width assigned to each domain. The domains exhibiting the high transition temperature undergo an evolution towards a well-defined state with a narrowing distribution of relaxation temperatures at the higher conversion values, whereas the low temperature relaxation is continuously extending over a wider domain. Comparing the NMR relaxation data as well as the calorimetric features of networks prepared by UV- or by EB-induced polymerization does not reveal noticeable differences to be related to the initiation mechanism (UV, EB or X-ray) and/or curing conditions (anisothermal or isothermal, dose rate). This was established for two undiluted aromatic diacrylates, but one should be careful and not generalize this finding to systems where mixtures of monomers are involved, since phase separation is likely to occur, hence inducing different reactivities in the segregated domains.

**Scheme 7:**
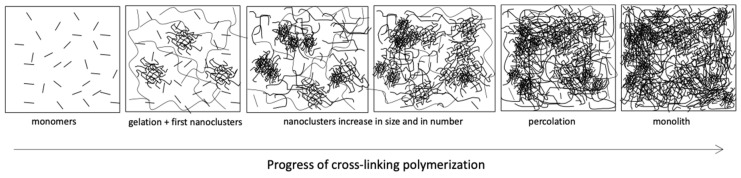
Sketch representing the heterogeneous build-up of networks prepared by radiation-induced chain polymerization of epoxy-diacrylate EPAC.

A common scenario accounting for these observations and measurements is proposed for the build-up of the network (Scheme 7). Irradiation of the liquid monomer induces the nucleation of softly interconnected gel nanoparticles within the swollen loose network, which will increase in number by additional nucleation, and in size by aggregation while the crosslink density goes up to form glassy nanoclusters. At a critical level, percolation of the nanoclusters induces syneresis of the material that ends up as a monolithic glassy solid. This illustrates variations of *T*_g_ and the broadness of the transitions in relation with the variety of defects and heterogeneities in the spatial distribution of cross-links density [138,141].

Research activities aiming at the improvement of matrix toughness, at the reduction of the matrix cure induced shrinkage, and at the design of fiber surface functionality are in progress with significant results in each of these topics. Solutions for matrix toughening with higher processability and compatible with environmental considerations are under development. A next step will consist of aggregating the technological solutions developed for improving isolated aspects of curing, examined from the viewpoints of processing, of curing kinetics, polymer network performances, and fiber-matrix interactions. Encouraging results allow envisioning mass production of structural composites as well as functional materials by means of a reliable, cleaner, and more productive out-of-autoclave manufacturing [142].

## 3. Graft Copolymerization Induced by Ionizing Radiation

### 3.1. Radiation-Induced Grafting of Solid Polymers

Radiation-induced grafting (RIG) [143] allows a rapid functionalization of solid polymers which can be easily upgraded from laboratory to industrial scale. This allows for a variety of applications [144]. Over the last 5 years, the major applications remain in accordance to societal needs with the development of: adsorbents for depollution (removal of toxic metals [144,145,146,147,148,149,150,151,152], ammonia/ammonium species [153,154], atmospheric CO_2_ [155]); exchange membranes for fuel cell (anion [156,157] and proton [158,159], batteries or super capacitors [116]; functional fibers other than adsorbent applications (flame-retardant [160], antistatic and antibacterial [161] properties); and numerous applications are emerging in the field of renewable energies [162].

RIG brings a durable functionalization of solid polymers as it modifies the initial polymer chemistry by covalent bonding of functional monomers [163]. Not just electron beams but also other forms of ionizing radiation such as γ-rays, X-rays, plasma [164], and swift heavy ion (SHI) irradiation can generate free radicals in the polymer and subsequently initiate grafting reactions. Modification by the RIG has several advantages over the conventional chemical methods. First, it does not involve any hazardous reactants, nor does it release any toxic side-products. These mild conditions required for grafting are appropriate for sensitive biopolymers and favors advances in green chemistry. Furthermore, by playing with the radiation penetration depth (energy range of incident particles), one can treat any polymer shape (films, fibers, nanoparticles, membranes, etc) thus modifying polymer surface or bulk. Commonly used monomers as styrene, acrylates, methacrylates, methacrylamides, vinyl acetates, and vinyl chlorides can be grafted.

The key parameter is the grafting yield (*GY*), which is determined as the weight of grafted copolymer with respect to the initial weight of polymer substrate:

(6) $$GY=\frac{m_{i}-m_{f}}{m_{f}}\cdot 100$$

where *m*_i_ and *m*_f_ are masses of the polymer before and after grafting, respectively. The grafting yield is mainly controlled by the irradiation exposure parameters such as dose, dose rate, and linear energy transfer (LET).

### 3.2. Radiation-Induced Grafting Processing

**Scheme 8:**
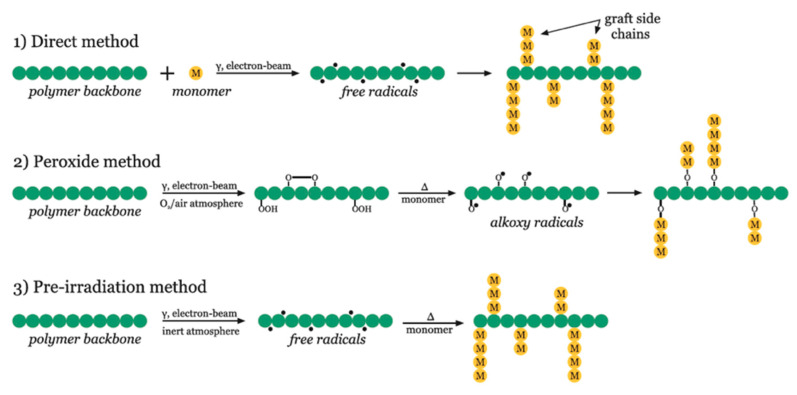
Scheme of radiation-induced grafting methods for polymers.

In total, three methods can be used for RIG: direct (mutual), peroxide, and preirradiation methods (Scheme 8).

In the direct method, the polymer substrate is immersed in a monomer solution and exposed to ionizing radiation. The irradiation activates the polymer substrate by the formation of free radicals. The free radicals of the polymer backbone initiate polymerization reactions of the monomer on its surface. After the initiation, the propagation of monomer chains takes place. The process continues until the growing macroradicals meet each other (recombination) and thus terminate the chain growth. Since the monomer solution is also exposed to irradiation, it may result in homopolymerization in solution limiting the *GY* value. For this reason, inhibitors or radical scavengers are generally added in the monomer solution.

In the peroxide method, the polymer substrate is first irradiated under oxygen or air atmosphere to form peroxides or hydroperoxides due to oxidation of alkyl radicals. At high temperature, the peroxide bonds decompose on alkoxy and hydroxy radicals. The alkoxy radicals initiate the polymerization reaction. In this method, which represents a special case of preirradiation method, the formation of homopolymer is negligible since the monomer is not exposed to irradiation.

In the preirradiation method, irradiation is carried out in vacuum or in an inert atmosphere to prevent radio-oxidation reactions. In contrast to the peroxide method, which involves peroxides/hydroperoxides decomposition, the alkyl radicals are reactive enough to themselves initiate the radical polymerization. To do so, a high concentration of radicals should be achieved, and high dose rates are required. Depending on the monomer reactivity, an activated temperature is generally needed. To avoid any radical quenching due to oxygen, the polymerization reaction should also be run under inert atmosphere and monomer solutions are de-aerated prior to RIG. It is worth noting that, under these precautions, the preirradiation method leads to covalent C–C bonding between the polymer substrate and the grafted polymer chains. At industrial scale, the preirradiation method makes the processes, i.e., irradiation and grafting, separable. Irradiated film rolls and bobbins of fibers are thus treated in continuous and batch modes [150].

**Figure 8:**
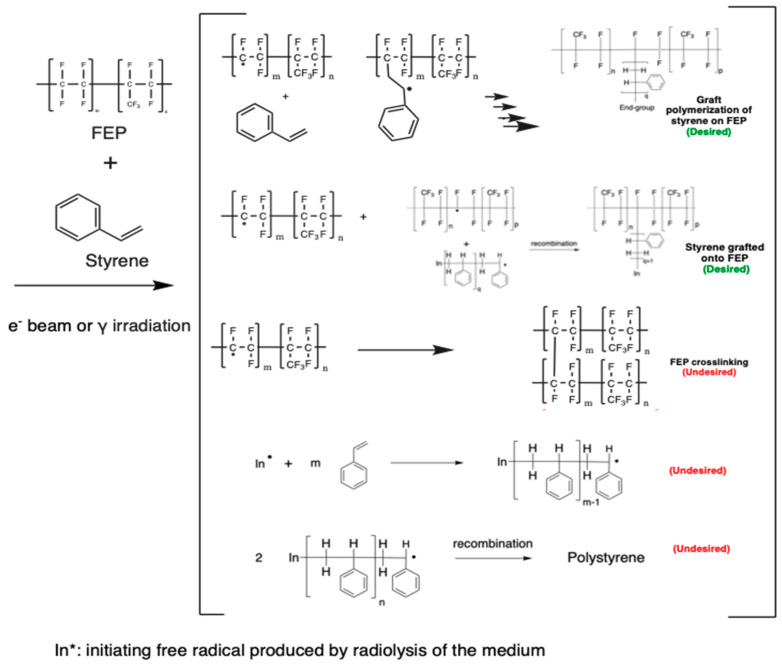
Possible chemical reactions between fluorinated ethylene propylene (FEP) and styrene during direct and indirect irradiation. The radicals produced will undergo propagation resulting in the polymerization of styrene. Undesired homopolymerization and FEP crosslinking may also take place [165].

A selection of possible reactions between styrene and fluorinated ethylene propylene (FEP) during direct and indirect irradiation is shown in Figure 8. Both the styrene and FEP are expected to form C-centered free radicals. However due to the high electronegativity of fluorine, it is expected that the FEP will produce higher yields of radicals and at a quicker pace than styrene. The desired products are those with increased covalently bonded styrene onto FEP while the undesired products increase the homopolymerization and crosslinking. Al-Sheikhly and coworkers investigated the mechanism of the radiation grafting of styrene to FEP [165].

### 3.3. Parameters Affecting the RIG

The *GY* is dependent on many factors, such as the polymer chemistry, monomer reactivity, solvent, dose, additives (e.g., inhibitor of homo-polymerization), reaction temperature, and atmosphere [166,167]. Therefore, the yield of grafting process can be controlled by varying these reaction parameters.

#### 3.3.1. Irradiation Dose and Dose Rate

Radiation-induced grafting by the direct method is usually performed using γ-rays in contrast to the preirradiation method, where the electron-beam is preferred [164,168]. In general cases, the higher the dose, the larger the number of radicals formed and the higher the *GY* is expected. However, the increase of the *GY* as a function of dose may exhibit a nonlinear dependence. At certain doses, no further growth in *GY* is observed. In the direct method, this phenomenon may be attributed to the restriction of monomer diffusion due to the increased viscosity of grafting solution due to the above-mentioned homo-polymerization. In the preirradiation method, the decrease in *GY* may be explained by achieving the gel–dose threshold of polymer. Crosslinking under irradiation can hinder the monomer diffusion inside the polymer but also the propagation of the grafting front. The inverse tendency is observed for the *GY* as a function of dose rate [169]. High dose rates produce higher density of radicals that favors their recombination and formation of gel.

#### 3.3.2. Polymer Substrate Chemistry

Among many polymers whose surfaces are modified by RIG, the most commonly used polymers are polyolefins such as polyethylene (PE) [146,148,155], polypropylene (PP) [148], and with a new trend using recycled polyolefin waste (PPw) [145]. There are also a significant number of reports of using surface modification of polyamide (PA) [155,161,170,171], poly(ethylene terephthalate) (PET) [155,172,173], polyurethane (PUR) [174,175], fluoropolymers [154] such as poly(tetrafluoroethylene) (PTFE) [176,177] poly(ethylene-*co*-tetrafluroethylene) [156,178] and poly(vinylidene fluoride) (PVDF) [116,153,159,179,180,181], cellulose [182,183], and many biopolymers such as chitosan [149]. Novel organic surfaces based on graphene oxide [151] have also emerged as organic materials that can be grafted by radiation.

*GY* depends not only on the number of radio-induced radicals but also on their reactivity toward the initiation of vinyl or allyl monomers. Electron paramagnetic resonance (EPR) spectroscopy is often used to determine the radical nature and content in the irradiated polymer samples and help predict the behavior in the RIG (see section on ‘Nature and trapping of radicals’ below).

Additionally, the total amount of radio-induced radicals is indirectly affected by some functional groups that can either stabilize some of the radicals or quench them. For example, considering polystyrene (PS), polypropylene (PP), and polyethylene (PE) irradiated at the same dose, the concentration of radicals, stable at room temperature, is in the following order: PS < PP < PE. Under comparable conditions, similar relationships were found for *GY* of poly(acrylic acid) (PAA) onto these polymers using the direct method [184].

#### 3.3.3. Monomer Concentration

The monomer reactivity is influenced by the type of solvent used for grafting as it involves the monomer diffusion. In general, the higher the monomer concentration, the greater the *GY*. However, both *GY* and rate of grafting tend to level off at certain monomer concentration beyond which further increase causes sharp fall in both parameters. This decrease is often caused by the decrease in the monomer concentration and the diffusion rate in the grafting zone. In the case of fluoropolymers, e.g., PTFE, which barely swells in the grafting mixture, such decreasing effects are attributed to the suppression of the monomer diffusion by the increase in the viscosity of the grafting close to polymer surface [165]. This phenomenon is attributed to the grafting front mechanism (see section on ‘graft front mechanism’ below).

#### 3.3.4. Solvent

The nature of solvent determines not only the *GY*, but also the location of the grafting. If poor-swelling solvent is used, surface grafting is most likely to take place due to the slow-down in monomer diffusion. When good-swelling solvent is utilized, bulk grafting is highly favored and homogenous grafting is preferably obtained. For example, PAA grafting preferentially occurs in the volume of PVDF film when water is used as a solvent [181]. Whereas the use of a transfer agent or less polar solvent promotes surface grafting. Furthermore, properly chosen swelling solvent may affect not only the grafting efficiency but also the homogeneity of the grafted chains.

#### 3.3.5. Grafting Temperature

Increasing temperature enhances the RIG by changing the kinetics of the reaction [185]. In the peroxide method, decomposition of the peroxides leads to the formation of active sites that initiate polymerization reactions. In the direct method, heating of irradiated solution above *T*_g_ of polymer, enhances the mobility of chain segments, promoting the active sited migration to the surface and thus increasing the population of radicals involved in the grafting process. At the same time, elevated temperature decreases the viscosity of monomer solution, making the diffusion of monomer inside the polymer bulk easier.

#### 3.3.6. Presence of Inhibitor of Homopolymerization

In the direct method, in order to suppress the undesired homopolymerization, inorganic salts, such as iron (II) chloride, copper (II) chloride, copper (II) sulphate or ammonium iron (II) sulphate (Mohr’s salt) may be used [186]. After dissolution, metal ions play a role of hydroxyl radical scavengers deactivating ^•^OH radicals into inactive OH^−^ ions [187]. Therefore, fewer side reactions are involved in the system and more monomers are available for grafting. This practice was also extended to preirradiation method, notably in case of reactive monomers known to promote transfer reactions such as acrylates [181].

### 3.4. Grafting Front Mechanism

The first radiation-induced grafting of fluorinated polymer substrates was reported in 1962 by Chapiro [143]. The author reported that styrene and methyl methacrylate (MMA) could be grafted inside of PTFE films using direct method at a low dose rate. Since PTFE substrates are not swollen in the monomer solution, the term grafting front mechanism was proposed. Afterwards, the concept of grafting front was shown by studying styrene or acrylic acid monomers onto PTFE or PVDF not only by direct method but also by preirradiation method [181,188]. The grafting front mechanism was evidenced in PVDF electron-grafted with *N*-vinylpyrrolidone using differential interference contrast microscopy by Ellinghorst et al. [189]. This mechanism depends on various parameters among which are the solubility properties of monomer and graft polymer. A good solvent of both monomer and graft polymer is required. Monomer transport is thus possible by the swelling of the substrate polymer upper layer at the solid–liquid interface due to graft polymer growing chains and subsequent diffusion of monomer solution into the swollen zone. As a result of progressive diffusion of monomer through the swelling layers, the grafting front moves towards the interior of the film. A homogeneous distribution of the grafted copolymer across the film thickness can only be achieved when sufficient *GY* is obtained.

### 3.5. Radiation-Induced Grafting of Semi-Crystalline Polymers

Essentially, the polymers used as substrates for RIG have to meet certain requirements in order to produce efficient grafting with desirable and functional properties. These polymers have to possess an ability to easily generate free radicals upon exposure to ionizing radiation and high resistance towards radiolytic degradation. Preferably, they are hydrophobic materials with high thermal, chemical and mechanical stability. Both hydrocarbon (among them PE is frequently used) and fluorocarbon films have been used in RIG technology. Compared to hydrocarbon, the fluorocarbon polymers have remarkable thermal and chemical stability [190,191]. These properties arise because of the strong C–F bond and the large size of the fluorine atoms can shield the carbon backbone of polymers from chemical attack. However, while the C–F bond is much stronger than the C–H bond, the G values for radical formation on high energy radiolysis of fluoropolymers are roughly comparable to those of their protonated counterparts.

The G value is the number of chemical changes induced by a deposited energy of 100 eV. For example, some typical G values for radical formation using γ-rays under vacuum at ambient temperature are 0.14 for PTFE [192]; 2.0 for FEP [193]; 0.93 for PFA [194]; 3.3 for PVDF [195]. Besides the thermal and mechanical stability, these polymers have also shown the capability to produce highly stable radicals when exposed to irradiation.

### 3.6. Nature and Trapping of Radicals

**Figure 9:**
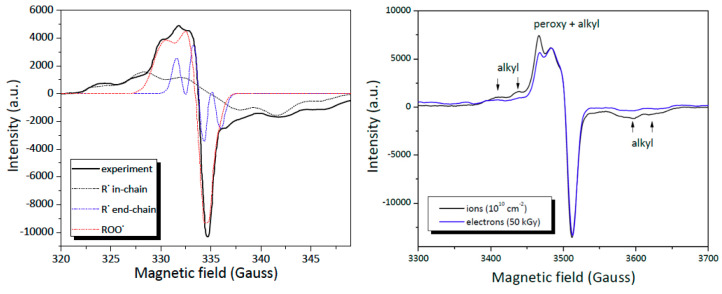
EPR spectra of 9 µm β-PVDF films irradiated by (**left**) e-beam (1.25 MGy); (**right**) Swift Heavy Ions (krypton of 10MeV/amu, fluence of 10^10^ cm^−2^ corresponding to 76 kGy) and e-beam (50 kGy).

The exposure to ionizing irradiation results in formation of alkyl, allyl, and peroxyl radicals in polymer. Regardless the type of irradiation, radiation-induced radicals in polymers have identical nature [196]. EPR spectroscopy is generally used for specification and quantification of the paramagnetic species. Signal amplitude (or integral intensity) increases with the increase of free radical content. At the resonance frequency and set magnetic field, g-value can be calculated. The g-value is used for identification of radical type. For example, the EPR spectra of irradiated β-PVDF films (Figure 9) show an overlap of characteristic signals of commonly radio-induced radicals in fluoropolymers [12,197,198]. There are various alkyl radicals formed in PVDF: –CH_2_ –C^•^F_2_ and –CF_2_ –C^•^H_2_ for end-chain alkyl ones; –CH_2_ –C^•^F–CH_2_ – and –CF_2_ –C^•^H–CF_2_ – for in-chain alkyl ones, and corresponding peroxyl radicals.

The EPR parameters of identified radicals consistent with [199,200,201,202] are collected in Table 1. Aymes-Chodur et al. have shown that alkyl radicals are the major species that induce PS grafting in γ-irradiated PVDF [12]. The carbon-centered radicals were mainly attributed to in-chain –CH_2_ –C^•^F–CH_2_ – and end-chain –CF_2_ –C^•^H_2_ radicals.

The ability of a polymer to trap and stabilize radiation-induced radicals in its structure is a crucial parameter for RIG, notably in the preirradiation method. In case of semicrystalline polymers, the crystalline fraction, remaining in the polymer after irradiation, significantly influences on radical trapping. At high dose, when the polymer is totally amorphous, no trapped radicals are observed in case of PVDF [203]. At lower doses, the radicals are trapped in zones driving different mobilities: crosslinked zones, crystalline and amorphous regions, interface of crystallites. Radicals trapped in inner polymer zones have less opportunity to react with monomer. Thus, depending on the location, (i) different diffusion rates are needed for monomer to react with radicals and (ii) some of the radicals may not be able to initiate grafting. Further work on PS grafting of SHI irradiated has shown that alkyl and peroxyl radicals are primarily located at the interface of crystallites and at the intra/intercrystalline amorphous zones of PVDF. This assumption was confirmed by FESEM observation that revealed the grafted PS network essentially localized on the spherulite lamellae [204].

### 3.7. Ion-Track Grafting

Despite the identical nature of radicals formed by ions and electrons in polymers, the distribution of energy deposition during irradiation is different. In the case of electron beam, the energy is randomly deposited in polymer substrate and grafted copolymer chains are homogeneously distributed. Contrarily, ions induce in their pathways continuous trails of excitations and ionizations leading to the formation of latent tracks. The deposited energy is highly localized along the ion trails; thus, heterogeneous grafting occurs.

Small-angle X-ray scattering (SAXS) was used to investigate the location of PS grafting depending on type of irradiation in α-PVDF [205]. It was found that the grafting in γ-irradiated polymer takes place in the amorphous zones of while the grafting is located in the latent tracks after swift heavy-ions (SHI) irradiation. Betz [206] made a detailed study of ion track grafting of MMA and styrene in α-PVDF. The following phenomenon was observed. When GY increases, the graft polymer spreads progressively around the track, destroying the crystalline structure in the bulk and covering the PVDF surface. It is worth mentioning that at high ion fluences (greater than 10^10^ cm^−2^), overlapping of tracks takes place. This may lead to radical recombination, which decreases the number of radicals and consequently lowering the *GY*. Nevertheless, even at high *GY*, SHI-induced grafting remained more heterogeneous than γ-induced grafting.

Similar to e-beam-induced grafting, one major application of ion-track grafting has been the development of fuel cell proton exchange membranes for automotive applications [207,208].

Whatever the irradiation source, high *GY* generally promote a mechanical degradation of polymer films. It is thus important to optimize obtained *GY* in irradiated polymers. A useful equation to convert the SHI fluence f into the adsorbed dose is the following:

(7) $$D=C\cdot f\frac{dE}{dx}$$

where *C* is a conversion constant equals to 1.6·10^−7^ J·mg·MeV^−1^·kg^−1^, *f* the fluence of incident ions and *dE*/*dx* is the stopping power (MeV·mg^−1^·cm^2^) [209].

### 3.8. RIG in Ion Track-Etched Polymer Membranes

Due to a high value of LET during SHI irradiation, narrow cylindrical damaged regions, so-called latent tracks, are formed inside irradiated polymer. The latent tracks are revealed by selective ion track etching in a highly oxidizing solution leading to nano-porous membranes. Nowadays, track-etched polymer membranes are used as filtration membranes and are commercially available. Several innovative applications are still being studied, notably their properties of acting as ionic diodes when asymmetrically etched are of greatest interest [210].

**Figure 10:**
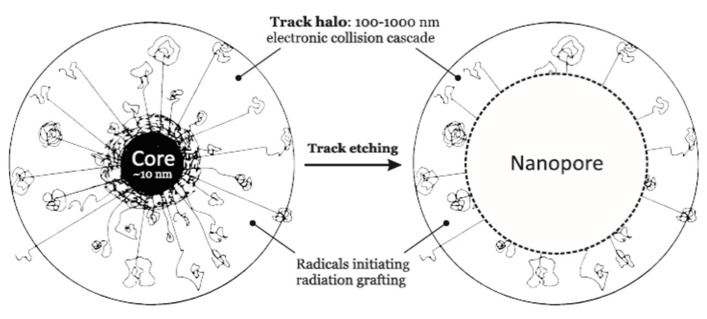
Schematic image of swift heavy ions (SHI) irradiated polymer film before and after etching.

Concerning RIG in track-etched polymer membranes, only a few groups thus far have reported on it. Recently, functionalization of nanopores of PET track-etched membranes was highly elaborated by Korolkov and Güven for the development of membranes by either using radicals trapped after etching or UV-induced grafting inside the nanochannels. The developed membranes were successfully used for direct contact membrane distillation of liquid low-level radioactive waste [211] as well as salt solutions [212]. PAA-grafted track-etched PET membranes were also employed as support to deposit gold nanoparticles to be used in catalysis [213]. The idea is based on the fact that the etching process removes only a part of polymer leaving many radicals inside the track nanopore wall (Figure 10).

**Figure 11:**
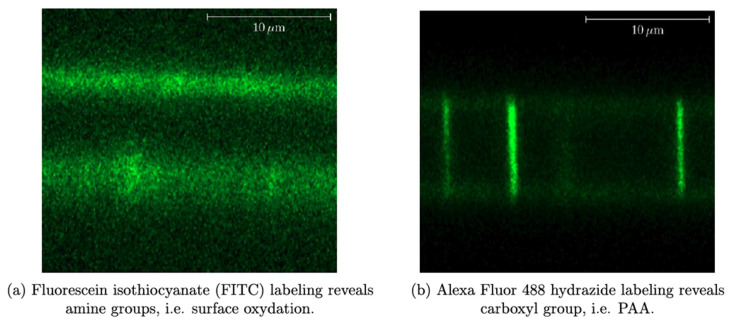
Confocal Laser Scanning Microscopy (CLSM) images of labeled PAA-g-PVDF membranes from ref [215].

In a peculiar polymer, namely PVDF, it was found that the radicals remained after etching were strong enough to initiate a RIG from the nanopore walls [214,215]. Due to the semi-crystalline structure of β-PVDF of about 40%, there is still a considerable number of radicals trapped in the crystallites, and at the interfaces between the crystalline and amorphous regions which are capable to initiate grafting reaction in presence of vinyl monomers. Mazzei et al. performed grafting of PS inside track-etched β-PVDF by means of SHI irradiation [214]. It was the first report assuming that the active sites remained after chemical etching were able to initiate grafting of styrene on the pore walls without using any supplementary irradiation source such as γ-rays or electron-beam. Confirming previous assumption, Cuscito et al. proved that, in the case of radiation-induced grafted PAA functionalities in track-etched β-PVDF (SHI of ^78^Kr 10 MeV/amu), the grafting location was solely inside the nanopores [215] (Figure 11).

Chemical etching results in formation of oxidized species, particularly carboxyl groups.

The presence of continuous fluorescence throughout the entire thickness of the membrane (Figure 11b) indicated that the grafting was performed homogeneously inside track etched PVDF. It also showed that the carboxyl groups of PAA remain chemically accessible and do not seem to be hindered by the confined environment of the nanopores. These results emphasized that the radical polymerization of acrylic acid monomer diffuse inside the PVDF polymer bulk as the grafting conditions were tuned to keep a surface grafting process. Further, 8 years later, the same group in France in collaboration with Güven’s team in Turkey succeeded to further exploit this remarkable property of β-PVDF track-etched membranes to tune nanopore size using RAFT-mediated controlled radical polymerization [216]. Many applications derived from these pioneer works are now available at prototype level for industrial needs such as sensors for toxic metal in waters [216,217,218,219].

**Figure 12:**
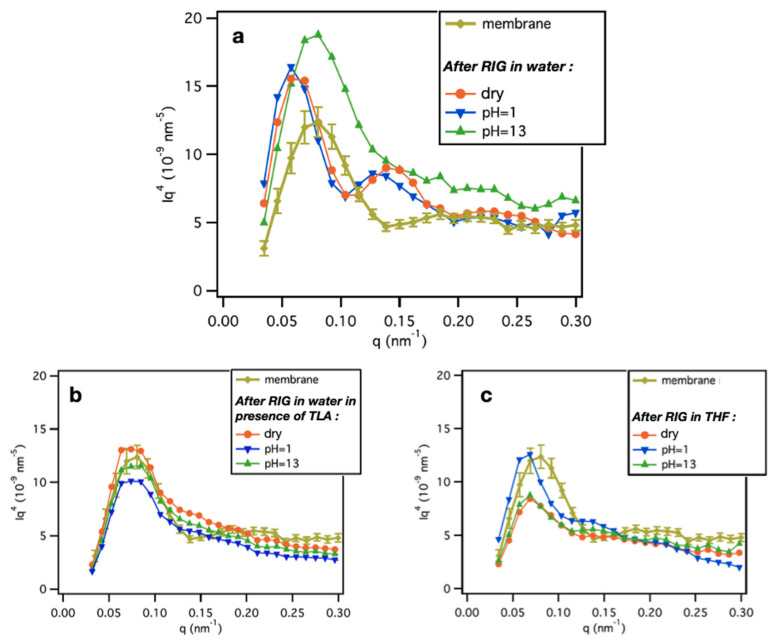
Small Angle Neutron Scattering (SANS) spectra obtained at LLB CEA Saclay, France (PACE spectrometer) for track-etched PVDF membranes exhibiting nanopores of 50 nm of initial radius (green circles), radiation grafted with PAA: (**a**) [AA] = 75% in water; (**b**) [AA] = 75% in water in presence of transfer agent, the thiolactic acid ([TLA] = 2.10^−3^ M) (**c**) [AA] = 25% in THF.

It is worth repeating herein that the general knowledge of RIG of solid polymers can be applied no matter the ionizing source of radiation. Typically, the nature of solvent affects the *GY* and coverage of etched ion-track walls similar to the corresponding e-beam-induced grafting on polymer surfaces. Figure 12 demonstrates very well the latter point as it shows the impact of a transfer agent such as the thiolactic acid or the use of less polar solvent such as THF on PAA grafting in etched ion-tracks PVDF membranes.

In each case, the radiation-grafted track-etched PVDF-g-PAA membranes were dried (red circle) and immersed in acidic (blue triangles) and basic (green triangles) media. It shows that, whatever the pH of the surrounded solution, the peak position corresponding to the nanopores diameter is not affected when surface grafting occurs at the nanopores wall (case of b and c). In the case of water (a), the PAA-grafted chains have penetrated inside the PVDF bulk, close to the surface, resulting in nanopore diameter changes due to various swelling of PAA grafted chains. It is worth mentioning that neutrons are only sensitive to high contrast of scattering diffusion length density meaning the interface of nanopore wall (pure PVDF or PAA-g-PVDF) with the solution or air.

## 4. Radiation Synthesis of Polymer-Based Nanogels

### 4.1. Nanogels

**Figure 13:**
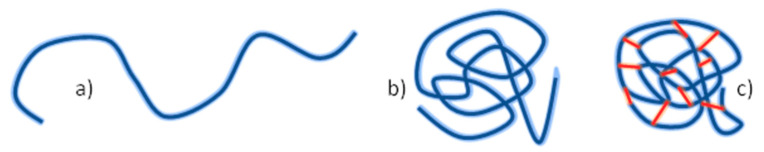
Nanogels as internally crosslinked polymer coils: (**a**) linear macromolecule in a stretched conformation, (**b**) linear macromolecule in a coiled conformation, (**c**) internally cross-linked polymer coil—a nanogel.

In the last two decades there is a growing interest in polymer-based nanogels as potential nanocarriers for controlled delivery of drugs, genes and radioisotopes, building blocks of scaffolds for tissue engineering, active components of biosensors, and other functional materials [220,221,222,223,224,225,226,227,228,229,230,231,232,233,234,235]. Nanogels can be defined as a particular topological form of macromolecules (Figure 13). In solution, flexible linear polymer chains typically attain the form of a coil. Such coils are dynamic structures, their segments being able to move (without breaking the chain continuity), and thus undergo spatial rearrangements. Additionally, such coils can easily unwind to stretched linear chains, what is observed e.g., if the polymer solutions are made to flow, thus the formation of coiled conformation is fully reversible. However, if additional “transverse” bonds are formed between the coil segments, the coiled structure becomes fixed and form a macromolecular cage, still soft and dynamic, but of nearly constant shape and dimensions. Such internally crosslinked polymer coils—nanogels, when made of hydrophilic and biocompatible polymers—are of considerable interest as carriers in biological environments and in this respect show some advantages compared to linear macromolecules.

One can also imagine a nanogel in an alternative way than the internally crosslinked single chain, namely as a small (nanosized) piece of a hydrogel network. By setting various synthetic conditions and inducing crosslinks either within one macromolecule or between many macromolecules, one can synthesize gels of any particle size from nanogels, via microgels up to macroscopic (wall-to-wall) gels. Patterns of micro-/nanoscale hydrophilic networks can also be fabricated on surfaces by deposition on pretreated surfaces the network precursors and inducing spatially controlled crosslinking reactions. In this chapter we will focus on nanogels as individual colloidal objects made of one or a few macromolecules.

Nanogels differ from linear macromolecules not only by topology. In solution they occupy lower volume than linear polymer chain of the same molecular weight [236], one of the practical aspects being lower solution viscosity and modified rheology, which can be of advantage for instance in paints and cosmetics formulations as well as in some applications as biomaterials [237,238,239,240,241]. Their conformation is more stable; while they do react to external stimuli by change in size, the amplitude of these changes is lower than for linear chains (see e.g., comparison of pH and ionic strength effect on poly(acrylic acid)—PAA—chains and nanogels in [242]). This may be of importance in biomedical applications (transport through biological membranes, endocytosis, etc.).

Furthermore, when compared to linear chains, nanogels are more resistant to degradation, irrespective of the cause of chain breakage. Any chain breakage event in a linear chain inevitably leads to disintegration and splitting up into shorter fragments. This is not necessarily so for nanogels, where each chain segment is typically connected to the others by more than one link. A breakage of a chain segment does not necessarily lead to disintegration and release of a chain fragment. This has been experimentally demonstrated for poly(*N*-vinylpyrrolidone)—PVP—as well as PAA nanogels and corresponding linear chains of these polymers subjected to oxidative degradation [242,243] and for hydroxyl-radical-induced degradation of neat and crosslinked hyaluronic acid [237].

### 4.2. General Synthetic Approaches

Nanogels can be synthesized by various methods. Actually, they are often formed as transient products or by-products in many polymerization and crosslinking procedures run in the bulk or in solution [243,244,245,246] (see Scheme 7 above and corresponding discussion), but such methods are not practical if nanogels are our main synthetic goal. The useful methods vary by kind of substrate (monomers vs polymers), phase composition of the reacting system (homogeneous vs. heterogeneous) and means of initiating the reaction (using heat, light, ultrasound, or ionizing radiation).

If we consider a mixture of monomers and crosslinkers (being typically bifunctional monomers) and initiate reactions in such a system, typically by generating free radicals, polymerization and crosslinking will occur side-by-side and the resulting product will be a crosslinked, three-dimensional polymer network. Performing such reaction in the bulk or in a homogeneous solution will usually lead to the formation of a macroscopic network (called a “wall-to-wall” gel, since typically the crosslinked product occupies the whole volume of the reaction vessel). But if our aim is to synthesize a crosslinked polymer structure of the size of a single coiled macromolecule (usually not more than 100 nm when in solution), we have to run the reaction in suitable “nanocompartments”, i.e., in micelles. Since we are mostly interested here in hydrophilic nanogels, and thus the monomers, crosslinkers and the resulting polymer structures should be soluble in water, synthesis should be performed in reverse miniemulsion or reverse microemulsion, having small droplets of the aqueous phase dispersed in the continuous oil phase [247,248].

In order to initiate crosslinking polymerization in such systems, one may use either a thermally activated polymerization initiator, or alternatively a photoinitiator, or apply ionizing radiation that allows for direct, initiator-free generation of radicals [249,250]. Such reactions can also be induced by ultrasound, with or without an initiator. Ultrasound may also be used to facilitate dispersion of components [251,252].

Such a synthetic approach, while effective, also has some drawbacks. It requires the use of many chemicals, generates waste, and is a multistep procedure; the products must be isolated from the oil/water system and purified. Unreacted monomer and crosslinker must be subsequently removed, in particular for when the product is intended for biological or medical use. Initiators and sometimes also surfactants may be embedded in the final product structure, which is often an undesired effect. Moreover, thermal decomposition of an initiator is a process difficult to be precisely controlled thus, also the product properties (e.g., crosslink density) are not easily controlled. Some of these deficiencies can be nowadays alleviated by using controlled radical polymerization techniques (CRP, [113,253,254]). In particular, the reversible addition–fragmentation chain transfer (RAFT, [255]) has been demonstrated to be very useful in controlling radiation-induced polymerization and crosslinking processes [256,257,258].

**Figure 14:**
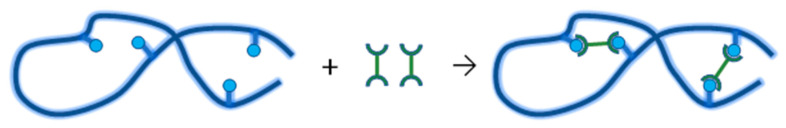
Intermolecular crosslinking of a macromolecule using a chemical cross-linking agent. For details see e.g., [259].

Another way to synthesize nanogels is by using ready linear polymer as a substrate and performing intramolecular crosslinking by utilizing the functional groups contained in the macromolecular structure. Most often this approach requires a bifunctional cross-linking agent having at least two groups capable of reacting with functional groups of the polymer (Figure 14). This reaction can be performed in homogeneous aqueous solution. An example may be internal crosslinking of poly(vinyl alcohol) chains by glutaraldehyde [259]. Care must be taken to optimize the reaction conditions, mainly the substrate and crosslinker concentrations, to maximize the yield of intramolecular crosslinking while limiting the extent of crosslinking between individual molecules. This method allows to eliminate the use of the oil phase, surfactant and initiator, as well as to obtain the product not contaminated by unreacted monomer. However, still purification steps are required to remove the rests of the crosslinking agent. Moreover, not all hydrophilic polymers of interest can be easily internally crosslinked in this way.

### 4.3. Synthesis of Nanogels by Radiation-Induced Intramolecular Crosslinking of Polymers

In order to further simplify the synthesis of nanogels and avoid the use of crosslinking agents, in the end of 1990, an alternative method utilizing ionizing radiation has been proposed, based on radiation-induced intramolecular crosslinking of macromolecules [260], for recent reviews see [236,261]. The only substrates are the hydrophilic polymer and water; neither initiators nor any other chemicals are used (except in some special cases). This method is described in some detail below. It builds upon the accumulated knowledge of radiation chemistry and in particular radiation crosslinking of polymers. 

#### 4.3.1. Radiation Chemistry of Polymers in Aqueous Solution

Ionizing radiation is relatively widely used in industry. Applications range from simple devices to measure the thickness and detect potential imperfections of products in steel industry to sterilization of medical devices and technologies where radiation is used to synthesize or modify materials. Commercial, large-scale irradiation facilities operate in many countries on all inhabited continents.

Ionizing radiation is defined as any kind of radiation (either photons or beams of accelerated particles) having enough energy to ionize atoms and molecules. In radiation processing, mostly two kinds of radiation are used: gamma rays and electron beams. Most large-scale gamma irradiation facilities use a radioactive isotope of cobalt, ^60^Co, as the source of gamma rays. Due to its metallic form, cobalt is relatively easy to process, handle, and store, while the gamma rays emitted by ^60^Co are high energy (average energy of 1.25 MeV), providing high penetration depth in matter and thus allowing to irradiate bulk materials (e.g., on standard palettes). Alternatively, electron beams (EB) generated by accelerators are applied.

In describing the action of ionizing radiation with matter, three important parameters are used. The dose is the amount of energy absorbed per unit mass of matter (in J/kg, which in radiation chemistry is called a gray—Gy), the dose rate is the dose absorbed per unit time (Gy/s) and the radiation–chemical yield (abbreviated simply as yield) is the number of moles of products or reactants (molecules, radicals, cross-linking bonds, etc.) produced or consumed per unit of absorbed energy (mol/J). Basic information on radiation chemistry and technology can be found in a number of books and reviews [262,263,264].

Electron beam technology provides high throughput due to high dose rates and does not require handling of radioactive isotopes, but, on the other hand, due to the limited penetration depth of fast electrons in matter, is less suitable for irradiation of bulk materials, typically requiring that the goods to be irradiated are packed in flat boxes or trays. Gamma rays offer high penetration in matter, allowing large boxes or whole pallets of goods to be irradiated, but the dose rates are lower and the isotope sources require proper handling and disposal. A new technology, X-ray conversion, combining the advantages and limiting the weak sides of gamma treatment and EB, is now being launched, and, when technical and economic aspects are improved, it will probably find its place on the market [265,266,267].

In general, polymers can be irradiated either in the solid state (i.e., in the bulk), or in solution, either in organic or aqueous solvents. Due to the desired hydrophilic character of the nanocarriers discussed in this chapter, our discussion will be focused on water as the solvent. The mechanisms and kinetics of reactions taking place in irradiated aqueous polymer solutions depend primarily on polymer concentration. The fraction of energy of ionizing radiation that is absorbed by a given component of the irradiated system is proportional to the electron density ratio of this component, which can be well approximated by the weight ratio. If polymer makes up a considerable fraction of a polymer/water system, a large part of the energy is directly absorbed by the polymer. While there are some important practical examples of such cases (irradiation of highly hydrophilic polymers which, for practical reasons, are not completely dry when irradiated [268] or irradiation of concentrated polysaccharide solutions, in the so-called paste state, to obtain macroscopic gels [269,270]), most of basic studies and applications are based on irradiating polymers in dilute or semi-dilute aqueous solutions. In dilute solutions, the direct action of ionizing radiation on the polymer itself is of minor importance and can be neglected in mechanistic considerations. It means that almost all energy is absorbed by water, and then the reactive products of water radiolysis can in turn attack the macromolecules, thus inducing reactions in the polymer component of the system. So, the polymer is subjected to an indirect effect of irradiation.

Radiation chemistry of water has been the subject of intense and detailed studies for over a century. Current state of knowledge on this topic can be found in relevant monographs [262,263,264,271], while comprehensive reviews provide detailed kinetic data on the reactions involved [272,273]. Here we only recollect the most basic facts relevant to nanogel synthesis.

Interaction of gamma rays or high-energy electrons with water leads to ionization and excitation of water molecules, and subsequently the cascade of very fast reactions results in the formation of transient radiolysis products, which can subsequently attack molecules of dissolved substrates. For millimolar concentrations of the latter (in case of polymers this refers to the concentration of monomer units), reactions with polymer chains occur on the timescale of nanoseconds and longer. 

The most important transient products of water radiolysis, in the absence of oxygen, are hydroxyl radicals ^•^OH, hydrogen atoms H^•^, and hydrated electrons e_aq_^–^ (Equation (8)), their radiation-chemical yields being ca. 2.9 × 10^−7^, 0.6 × 10^−^7, and 2.9 × 10^−7^ mol/J, respectively. In laboratory conditions or small-scale synthesis, hydrated electrons can be easily converted into further hydroxyl radicals in a reaction with nitrous oxide, which is highly soluble in water (Equation (9)).

H_2_O → e_aq_^−^, ^•^OH, H^•^, H_2_O_2_, H_2_, H^+^, OH^−^ (8)

N_2_O + e_aq_^−^ +H_2_O → ^•^OH + OH^−^ + N_2_ (9)

**Figure 15:**
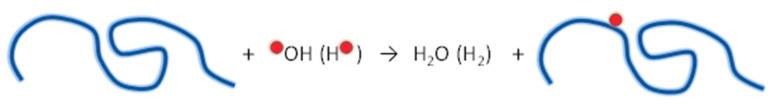
Formation of randomly-located carbon-centered polymer radical as a result of hydrogen abstraction by hydroxyl radicals or hydrogen atoms from a polymer chain.

The most common reaction of water radiolysis products with simple aliphatic polymers is hydrogen abstraction from C atoms by ^•^OH radicals and by H^•^ atoms. As a result, a radical is formed at the carbon atom of the polymer, usually at a random position along the polymer chain, with concomitant formation of water or hydrogen molecule (Figure 15).

**Figure 16:**
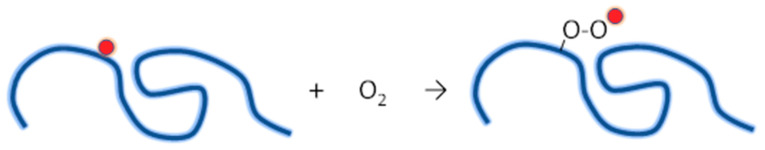
Addition of oxygen molecule to a carbon-centered polymer radical leading to the formation of a peroxyl radical.

Now we should briefly consider the typical reactions of these radicals. If oxygen is present in the solution, it reacts with the polymer alkyl radicals forming peroxyl radicals (Figure 16). This subsequently leads to complex chemistry [274,275] involving chain oxidation processes, decomposition of peroxyl radicals via unstable tetroxides, formation of carbonyl groups, etc. Since these processes usually do not lead to crosslinking, at least in the sense of formation of stable covalent bonds between polymer chains and/or chain segments, irradiation in the presence of oxygen is undesirable. However, under conditions that are favorable for nanogel synthesis (i.e., low polymer concentration and N_2_O-saturated solutions), oxygen is produced during irradiation in minor amounts. (see [241,242,276]).

Usually in radiation processing of polymers the desired reaction is crosslinking, as discussed in more detail below. However, one should be aware of the presence of other reactions that may influence the outcome of irradiation, or even dominate the radiation chemistry thus preventing the polymer from being crosslinked. In general, we may divide radiation-induced reactions in polymers into two groups, i.e., processes involving one radical (mainly H-shift, degradation and addition to a multiple bond; in these processes the number of radicals in the system does not change) or involving two radicals (crosslinking, disproportionation; as a result of these reactions radicals decay).

**Figure 17:**
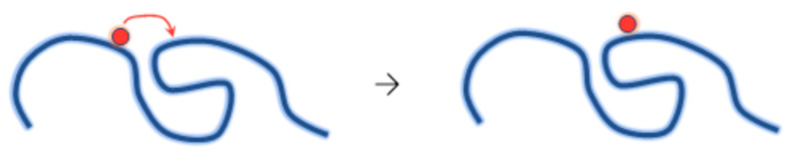
H-transfer leading to a shift in location of the radical along a polymer chain.

**Figure 18:**
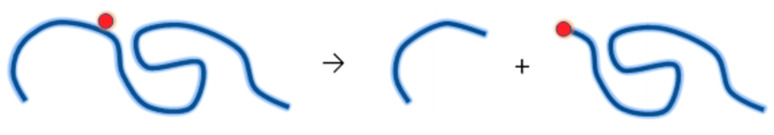
Degradation (chain scission) of a radical-bearing macromolecule.

**Figure 19:**
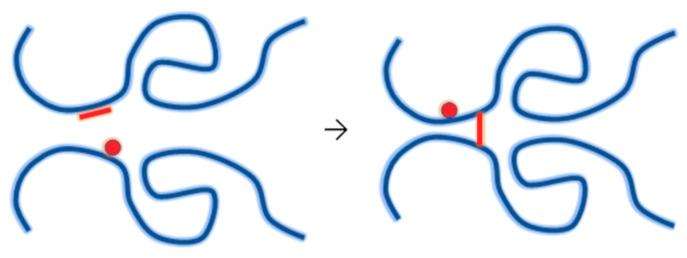
Intermolecular addition of a polymer radical to a pre-existing double bond, resulting in crosslinking and re-creation of a radical site.

In principle, H-shift (Figure 17) seems to be a benign process apparently not influencing other reactions in the system, since the number of radicals on polymer molecules remains unchanged. Moreover, such reactions have been shown to be relatively slow [277,278]. However, H-shift has been demonstrated to have some impact on the kinetics of intramolecular recombination, due to its influence on spatial distribution of radicals along a polymer chain [279]. Degradation (chain breakage, Figure 18) is a common process in radiolysis of polymers. As discussed in detail above, for some macromolecules (nonmodified polysaccharides, aliphatic polymers containing tertiary carbon atoms), when irradiated at standard conditions, it can be the sole or at least the dominating reaction. Even in the case of polymers undergoing predominantly crosslinking, often some contribution of degradation can be observed [8]. Thus, it is considered as an important process that competes with crosslinking and always should be taken into account when studying radiation effects on polymers. In particular cases, chain breakage and the consequent formation of a terminal polymer radical can initiate chain process of depolymerization [280]. In case the macromolecules in question contain double carbon–carbon bonds (for instance formed as a result of previous disproportionation or degradation steps), a polymer-free radical may add to such a bond, forming an inter- or intramolecular crosslink (Figure 19).

**Figure 20:**
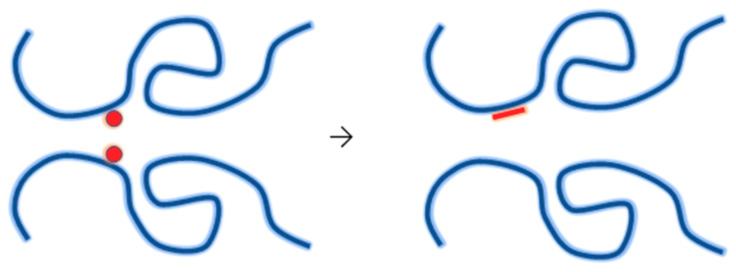
Intermolecular disproportionation of two carbon-centered polymer radicals.

The bi-radical reactions are recombination (crosslinking) and disproportionation. The ratio between the two processes is mainly governed by chemical structure of the polymer and cannot be easily influenced (for an exception where this ratio can be to some extent controlled by pH due to the presence of carboxyl groups at the radical-bearing chain, see [281]). Disproportionation (Figure 20; the process can also occur intramolecularly) is often neglected as an unwanted side process in polymer crosslinking. However, it should be kept in mind that in some cases the yield of disproportionation may actually be much higher than the crosslinking yield, leading to considerable loss of radicals and limitation of the crosslinking yield; a good example is poly(vinyl alcohol) where ca. 90% of radicals disproportionate [282].

**Figure 21:**
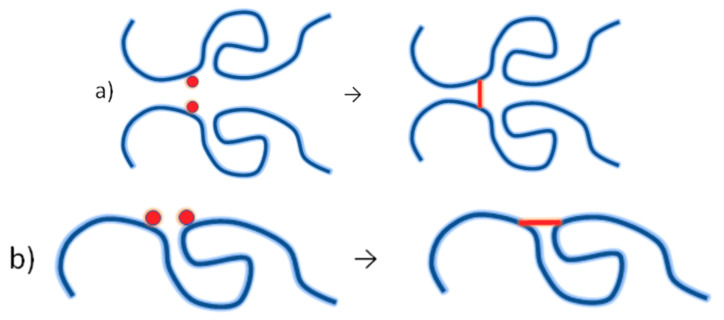
Crosslinking by recombination of two polymer radicals: (**a**) intermolecular, (**b**) intramolecular.

Radical recombination, leading to polymer crosslinking, can occur in two ways, either between two radicals located at separate macromolecules (Figure 21a), or between two radicals present at two different segments of the same chain (Figure 21b). The former leads to covalent binding of the two chains and an increase in the molecular weight. Consequent acts of intermolecular crosslinking may finally lead to the formation of macroscopic, three-dimensional polymer networks, where most or all the chains present in the system are linked together. This reaction is the basis of many implemented technologies: reinforcing of tires, tubes, cable insulations and jackets, production of thermoshrinkable materials or improving wear resistance of sockets of hip joint implants when polymers are radiation-crosslinked in the solid state, or hydrogel wound dressings and drug delivery systems [283,284] when crosslinking is performed in aqueous solution. In contrast, when intramolecular crosslinking occurs within a single macromolecule, a nanogel particle is formed.

#### 4.3.2. Synthesis of Nanogels by Intramolecular Crosslinking

**Figure 22:**
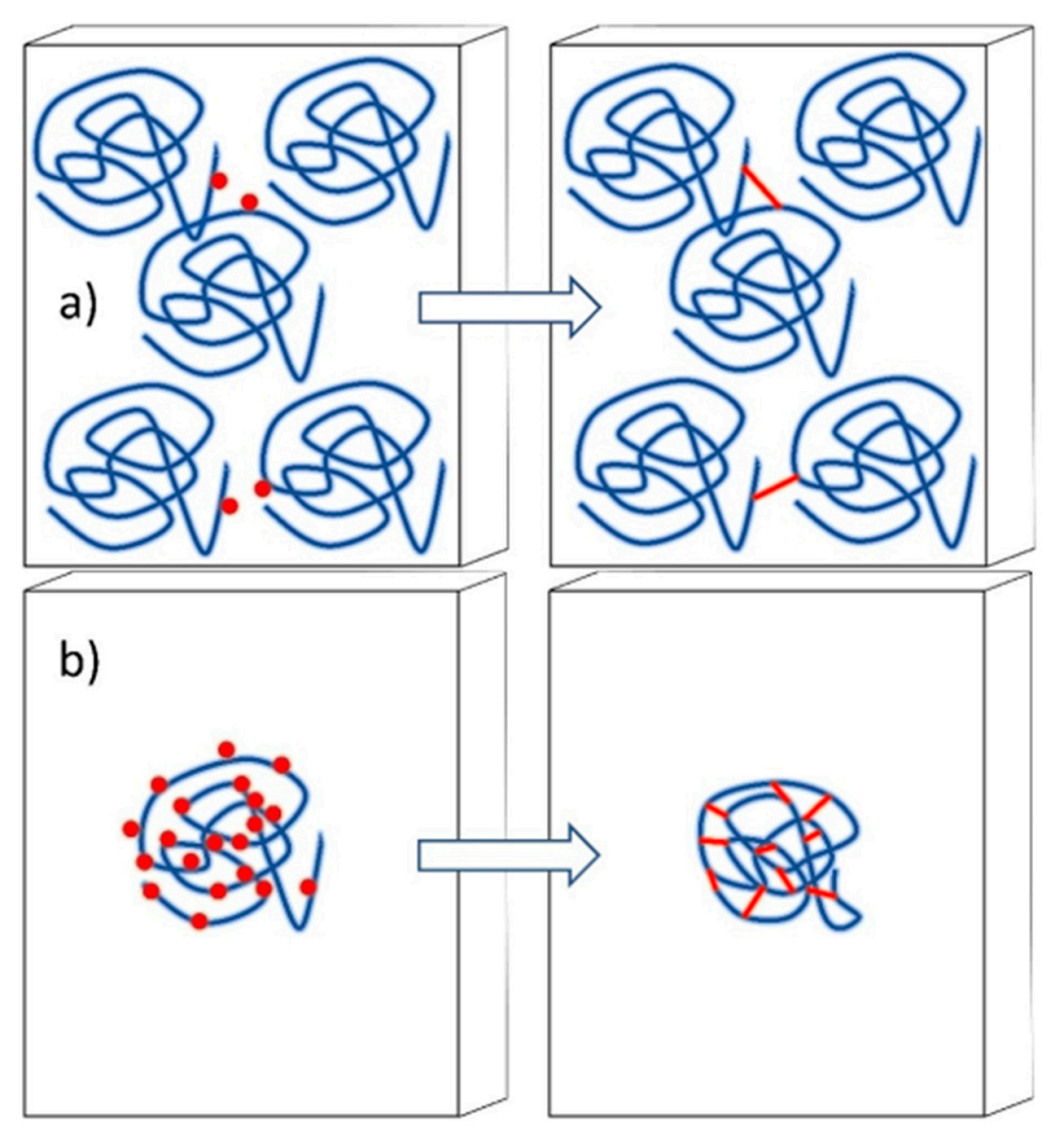
Controlling the dominating crosslinking process: (**a**) high polymer concentration and low dose rate—n average less than one radical present simultaneously on each chain—domination of intermolecular crosslinking, (**b**) low polymer concentration and high-dose pulse irradiation—many radicals present simultaneously on each chain—domination of intramolecular crosslinking.

The competition between inter- and intramolecular recombination of polymer radicals can be controlled by reaction conditions (Figure 22). The key factors to achieve this control are the average number of radicals that are present simultaneously on each polymer chain and the concentration of radical-bearing polymer chains. When discussing this issue, one should bear in mind that radicals are very reactive species thus, except special cases (see [277,285,286]), in solution they disappear within a fraction of second after being formed. If we irradiate a moderately concentrated polymer solution (typically a few weight %) in a continuous way using a relatively low dose rate (as would be the typical conditions for gamma irradiation), the rate of radical decay will soon approach the rate of radical production, and a steady-state is established where the average number of radicals per chain may be significantly less than one. If so, the probability of intramolecular reactions is very low (since it is very improbable to find 2 or more radicals simultaneously on the same chain) and the radicals would have to find reaction partners on other macromolecules (Figure 22a). In contrast, if we use a dilute solution (typically below 1%) and deliver radiation energy in short, intense pulses (which is the typical mode of irradiation when using electron accelerators), it is easy to generate many radicals (even many tens of them) on each chain simultaneously, within the pulse duration, which may be as short as nano- or microseconds. Since, due to low polymer concentration, the next chains are far away and their diffusion is slow, and chances for intermolecular recombination are low (albeit not zero, see below), while most of the radicals would recombine with their neighbors on the same chain (Figure 22b). In this way nanogels are formed.

**Figure 23:**
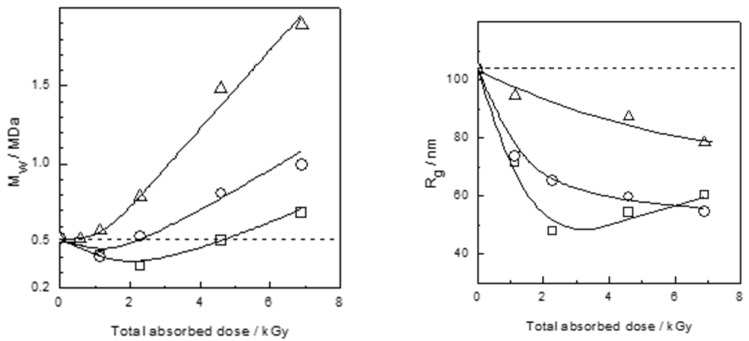
Radiation synthesis of poly(acrylic acid)—PAA nanogels. Weight-average molecular weight (*M*_w_—left panel) and radius of gyration (R_g_—right panel) of PAA macromolecules in the course of nanogel synthesis as a function of total absorbed dose (1.15 kGy corresponds to a single pulse) for samples of various PAA concentrations: ◻—10 mM, ○—17.5 mM, △—25 mM, irradiated in Ar-saturated aqueous solutions, pH 2. Radii of gyration measured at 25.0 °C in aqueous 0.5 M NaClO_4_, pH 10. Reprinted with permission from [240]. Copyright (2003) American Chemical Society.

The characteristic feature of radiation synthesis of nanogels is a significant decrease in coil dimensions (radius of gyration, R_g_) while the average molecular weight (*M*_w_) remains nearly constant or slightly increases (since it is difficult to totally eliminate intermolecular crosslinking). Decrease in dimensions reflects the formation of internal bonds, which limit the ability of segments to diffuse away from the center of mass of the macromolecule (for illustration, see Figure 23b below). This is also a typical feature observed in chemical intramolecular crosslinking [259]. In some cases, the pattern of R_g_ and *M*_w_ changes becomes more complex. For poly(acrylic acid) initially some decrease in *M*_w_ is observed, due to degradation competing with crosslinking. However, when some internal bonds are already formed, further degradation acts do not lead to detachment of chain fragments and thus they do not cause any further decrease in molecular weight. Since some intermolecular crosslinking is present, the *M*_w_ starts to increase, while the dimensions go down (Figure 23). It can be further seen that the competition between intra- and intermolecular crosslinking can be influenced by polymer concentration, since this, at a constant dose per pulse, influences the average number of radicals per chain.

Nanogel synthesis can also be performed on industrial electron beam installations with high throughputs, such as those used for sterilization purposes, with no modification to the regular irradiation procedures [287,288]. Solutions, contained in sealed vials, are irradiated within a box passing under the scanner on a moving conveyor. The electron pulses are modulated with two frequencies; the frequency of the accelerator and the frequency of the scanning horn at which the beam sweep across the width of the samples box. The total adsorbed dose is also controlled by speed of the conveyor belt. Within certain limits, the nanogel size and molecular weight can be fine-tuned by changing the polymer concentration. At low polymer concentration, intramolecular crosslinking is the dominating process that leads to nanogel formation. At higher polymer concentrations, intermolecular crosslinking is initially competitive with intramolecular crosslinking but, through this process, the polymer concentration decreases with dose to the point where intramolecular crosslinking becomes dominating [276]. Beyond this point, no change in molecular weight or size is observed. The increase in size and molecular weight generally occur well below 20 kGy [241,242]. Higher doses are imparted by multiple passes. When a high dose range is explored (20 kGy to 80 kGy), e.g., with N_2_O-purged poly(*N*-vinyl pyrrolidone) solutions (concentrations in the range 0.05–0.2 %w), the average molecular weight and hydrodynamic size of the produced nanogels do not significantly change with increasing the dose but low molecular weight fractions appear at higher doses. New functional groups, such as primary amino groups and carboxyl groups, only to mention the most interesting ones for postirradiation reactions, are formed in a dose-dependent fashion [241,242,288]. Both experimental measurements and numerical simulations suggest that O_2_ is also formed under these irradiation conditions. In fact, irradiation of a polymer in dilute aqueous solution by high-dose pulses of electrons causes some hydroxyl radicals to recombine rather than react with the polymer, and in this way substantial amounts of hydrogen peroxide are formed. Under high-dose irradiation, some of the formed hydrogen peroxide is converted in molecular oxygen. The reaction of molecular oxygen with polymer radicals is at the basis of the observed dose-dependent polymer fragmentation and network functionalization. As mentioned above, double bonds are also formed in radical-radical disproportionation reactions [242,276].

**Figure 24:**
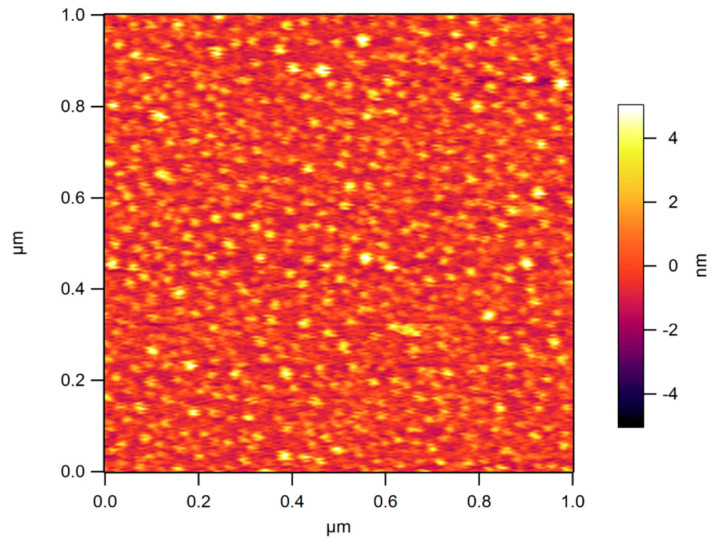
Height profile from tapping mode AFM under water for radiation-synthesized PVP nanogels modified with acrylic acid. The color scale represents a maximum height of 8 nm. Reproduced from [292].

**Figure 25:**
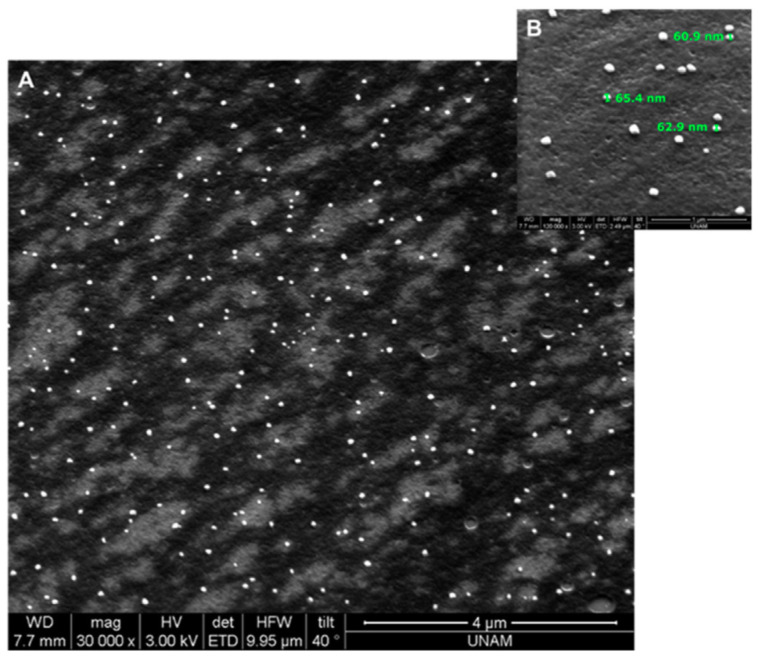
The scanning electron micrographs of PVP nanogels at two different magnifications (**A**,**B**). Reproduced from [291]. License obtained from Elsevier.

Nanogels can be visualized by AFM and SEM microscopy when adsorbed at a flat surface [240,289,290,291]. Figure 24 illustrates PVP nanogels grafted with acrylic acid, as scanned by AFM in the tapping mode under water, while exemplary SEM pictures of PVP nanogels cast on a silicone surface and sputtered with gold are shown in Figure 25.

Intramolecular recombination is an interesting process from the kinetic point of view and has been the subject of numerous experimental and simulation-based studies [240,276,279,289,293,294,295,296]. When analyzing the general decay of radicals in the system it has often been observed to deviate from the classical second-order kinetics typical for recombination of radicals located on small molecules. Both experimental data and Monte–Carlo simulations indicate that the spatial distribution of radicals along the chain is the decisive factor both for kinetics and the final structure of the nanogels [279,294]. A dispersive kinetics model developed by Plonka [297,298] is helpful both for kinetic description and mechanistic interpretation of intramolecular radical recombination. Alternatively, for every pair of radicals defined by a given combination of radical positions, a rate constant can be defined. In a recently developed modeling approach, intramolecular radical-radical reactions were treated on the basis of combinations of site-dependent rate constants. This model could reproduce the kinetics of radical decay in single-pulse experiments [296] as well as the evolution in molecular weight in nanogel synthesis using pulsed e-beams [276]. As this model took the complete set of radiation-induced reactions into account, essential information about the evolution of other species in the system could be derived. This includes the already mentioned formation of O_2_ as well as double bonds due to radical–radical disproportionation [276]. Recently, new kinetic studies using the Dynamic Liquid Lattice model [299,300,301,302,303] have been initiated on intramolecular crosslinking of macromolecules.

The above-described nanogel synthesis method is not limited to polymers of any particular chemical structure, albeit it requires a polymer which predominantly crosslinks, and not degrades, under irradiation. It has been successfully tested on simple synthetic water-soluble polymers—(poly(*N*-vinylpyrrolidone) [236,287,288,289,290,291,292,304,305,306,307,308,309,310,311] and poly(vinyl alcohol) [260], on an exemplary polyelectrolyte [poly(acrylic acid)] [240,312], and on a thermosensitive polymer—(poly(vinyl methyl ether)) [313]. When working with polyelectrolytes, one should set appropriate pH where ionization is suppressed. Otherwise the charges along the chain repel each other by Coulombic forces, which makes the energy barrier for recombination (and disproportionation) so high, that the radicals can live on chain segments for hours (at R.T., in water, with no stabilizing effects other than the presence of charge) [277,285,286], and slow reactions such as degradation dominate over recombination. For instance, for poly (acrylic acid) (average pK_a_ for high-molecular-weight polymer being ca. 6, [314,315]), crosslinking starts to prevail only when pH is lowered to 3 or less, and successful nanogel synthesis has been demonstrated at pH 2.

#### 4.3.3. Controlling the Physicochemical Properties of Radiation-Synthesized Nanogels 

While the feasibility of radiation-induced intramolecular crosslinking for nanogel synthesis is well established, recent studies have been focused on controlling the process, so that starting from macromolecules of given molecular weight and dimensions one could arrive at nanogels of desired *M*_w_ and R_g_ (which also determines the coil density of the final, crosslinked product).

**Figure 26:**
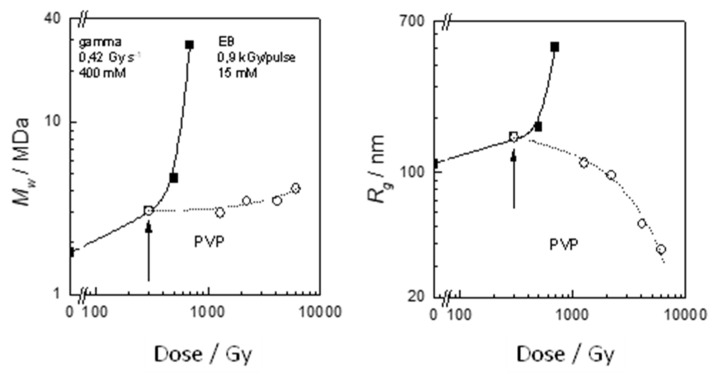
Two-step synthesis of PVP nanogels. M_w_ and R_g_ as a function of total absorbed dose. Black symbols denote continuous low-dose-rate gamma irradiation at a polymer concentration of 400 mM. In the two-step procedure, at the point marked by an arrow, irradiation conditions are changed—the second step (open symbols) is pulsed EB irradiation at the PVP concentration of 15 mM. Based on [305].

Kadłubowski et al. proposed a two-step synthetic procedure of synthesizing tailored nanogels [305]. In the first step irradiation conditions are set to promote intermolecular recombination (relatively high polymer concentration, moderate dose rate). As a result, both average molecular weight and the nanoparticle size increase with dose. When the desired average molecular weight is reached, the solution is diluted, and irradiation mode is changed to short, intense pulses. These conditions promote intramolecular recombination; therefore, the molecular weight does not significantly change, but the size is reduced to the desired value. Figure 26 shows the test of this approach for poly(*N*-vinylpyrrolidone).

An extensive study on the radiation synthesis of PVP nanogels has been recently published by Sütekin and Güven [291]. The effects of various parameters as total absorbed dose, dose rate, polymer concentration, molecular weight on the sizes of nanogels were investigated. It has been proved that by careful control of these parameters, PVP nanogels of sizes varying in the range of 30–250 nm can be prepared. It has been demonstrated that, when proper conditions are chosen, nanogels can also be synthesized using gamma rays. The nanogels have been shown to maintain their parameters for 2 years of storage in solution at 4 °C.

For polymers that undergo temperature-induced phase transition, for instance, those characterized by lower critical solution temperature (LCST), temperature can be used for controlling the crosslinking process. Pulsed EB irradiation of poly(vinyl methyl ether)—PVME—in dilute aqueous solution below the LCST (36 °C) leads to thermo-sensitive nanogels, which exhibit lower LCST (29 °C) than the parent linear polymer [316]. Irradiation of PVME solutions above LCST leads to the crosslinking of collapsed polymer aggregates, resulting in nanogels of different sizes and structures than those obtained by irradiation at low temperature [317].

**Figure 27:**
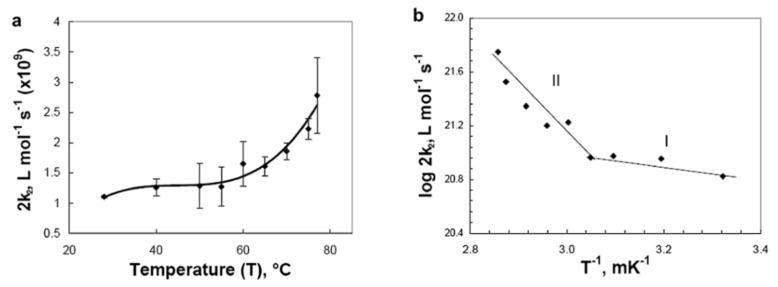
(**a**) Second-order reaction decay rate constant (2k_2_) as a function of temperature; (**b**) Arrhenius plot of 2k_2_ showing how the average activation energies were derived from two different temperature regions (I and II) in N_2_O-saturated PVP aqueous solutions [289].

An and co-workers have demonstrated that formation of PVP nanogels by pulse irradiation in aqueous solution can also be influenced by temperature [289]. Above a threshold temperature of 50–55 °C, due to the thermal collapse of polymer chains, probability of intramolecular recombination increases sharply and there is a strong change in the activation energy of radical decay, which leads to the formation of compact nanogels. The reaction rate constants of decay (2k_2_) were measured at temperatures ranging from 28 to 77 °C, Figure 27 shows their results. These results are not unexpected, as at higher temperature ranges, the distance between the carbon-centered radicals on a single change are smaller due to the polymer taking on a collapsed chain conformation. At low temperatures, the polymer chain is a noncollapsed random coil whose dimensions are closer to those predicted by random walk statistics. The carbon-centered radical–radical distances are also longer pointing towards a lower 2k_2_ values as observed.

In addition, An and co-workers also provide a mathematical model for determining the concentration of radicals on a chain to analyze the decay kinetics of the radicals formed. The calculations were based on a monodisperse PVP model. The spatial distribution of the polymer molecules was based on Poisson distribution of *Z* molecules in *N* cells as follows (Equation 10)

(10) $$P\left(m\right)={\left(\frac{Z}{N}\right)}^{m}\frac{e^{\frac{-Z}{N}}}{m}$$

where *P(m)* is the probability that a cell has exactly *m* polymer molecules [289].

To study the radicals generated and their reactions, the effective concentration has to be determined. This can be accomplished by taking the volume of reactive radicals aka the hydrodynamic volume into consideration. The effective molar concentration is then (Equation 11)

(11) $$C_{r}=\;\frac{1}{N_{A}V_{b}}$$

where N_A_ is Avogadro number and *V*_h_ is the hydrodynamic radius.

#### 4.3.4. Controlling the Chemistry of Radiation-Synthesized Nanogels

While nanogels made of homopolymers may be themselves interesting materials for various applications, and, in some cases—such as nanogels based on poly(acrylic acid)—can be easily functionalized, it is of considerable interest to adjust the above-described synthesis method to provide ability of chemical modification of the parent polymer, ideally already in the synthesis step. For many applications it would be of interest to obtain nanogels possessing some specific chemical functions, while having the main structure based on a well-tested and fully biocompatible polymer as PVP. Such functional groups can either render the nanogels some specific properties (e.g., surface charge) or allow for easy functionalization, for instance with drugs and/or targeting moieties needed to transform a nanogel into a targeted nanocarrier for controlled drug delivery. It has already been mentioned that such functionalization effect can be achieved, in some cases, just by careful adjustment of the conditions of the radiation synthesis itself, with no chemical additives [241,242].

One can also introduce chemical modifications to the nanogel structure by adding suitable chemicals, for instance monomers, to the polymer solution before irradiation. Monomer molecules can add to the polymer-derived radicals and either remain there as single-unit branches or they can initiate graft polymerization. If the monomer concentration is low, the branched chains will remain short. Other reactions of the added monomers are also possible, such as homopolymerization terminated by recombination with polymer-derived radical or by addition to the polymer double bond resulting from disproportionation. The product will contain short grafted chains and/or just single monomer units of the added monomer, providing the required functionality. In this way, PVP nanogels containing carboxyl groups can be synthesized by pulse-irradiation of PVP solution containing acrylic acid [225,292,308,318,319]. A similar approach has been demonstrated to yield poly (*N*-vinyl pyrrolidone)-graft-(3-*N*-aminopropyl) methacrylamide nanogels [287]. This indicates the great versatility of the radiation method for synthesizing nanogels of various physical and chemical properties, adjusted to the needs of particular applications, especially in pharmacy and medicine.

Another interesting option to obtain nanogels containing different chemical functions is by radiation crosslinking of interpolymer complexes. Such complexes may be based on various interpolymer interactions. One of the most interesting classes of interpolymer complexes are those based on hydrogen bonding [320,321,322]. They are formed by two polymers, one being a donor and the other an acceptor of hydrogen atom. As the donor, most often polymers bearing carboxylic groups are applied, while polymers possessing ether or carbonyl groups are usually used as counterparts. Hydrogen-bonded interpolymer complexes (IPCs) not only combine the chemical properties of the parent polymers, but also form heterogeneous structures containing hydrophobic domains, which may be useful for instance in solubilization and controlled delivery of hydrophobic drugs or drugs that have to be protected from low pH and enzymes in the stomach [323,324,325].

Hydrogen-bonded IPCs based on polymers with carboxylic groups are formed only below a specific, critical pH, pH_crit_. This may seem obvious, since at high pH the dissociated carboxylate groups lack the hydrogen atom capable of forming the H-bond. However, pH_crit_ is typically much lower than pK_a_ of the parent polyacid. This is because for the stable complex to be formed, an uninterrupted sequence of several protonated carboxylic groups must be present along the chain. For instance, average pK_a_ of poly (acrylic acid) is ca. 6 [315], while pH_crit_ for its complexes with poly(*N*-vinylpyrrolidone) is ca. 3.7–4.0 [321,325]. As a result, the complexes exist only at low pH, and dissociate in neutral and alkaline solutions. This instability at typical physiological pH may be perceived as a disadvantage in some applications. Radiation crosslinking, performed at pH < pH_crit_, in an analogous way to the intermolecular crosslinking of homopolymer chains described above, can be a simple and efficient way to “fix” the complexes and make them permanent. This, of course, does not prevent the complexed domains to become dissociated at neutral and high pH (albeit complexation in a crosslinked system may differ in some respects from complexation of two separate chains [326]), but it prevents the whole structure from disintegration. From the chemical point of view, the product is an internally crosslinked block copolymer. Crosslinking of PAA-PVP complexes, leading to the formation of permanent nanogels, by pulses of fast electrons in acidic aqueous solution has been described by Henke et al. [325,327]. This idea has been further developed by Güven et al. in their detailed studies on radiation crosslinking of PAA-PVP [328] and PAA-PEO complexes [329]. In the latter work it has been clearly demonstrated that by adjusting the solvent composition (acetone has been used as co-solvent) one can influence the size of the formed nanogels. An alternative way of synthesizing PAA-PVP nanogels has been developed by Abd El-Rehim and co-workers. Gamma irradiation has been used to initiate template polymerization and crosslinking in aqueous solutions of PVP and acrylic acid [330,331,332,333].

### 4.4. Biomedical Applications of Radiation Engineered Nanogels

The success of nanogels is mainly due to their promising applications in the biomedical field. Nanogels, as stand-alone nanoparticles, have been proposed as nanocarriers of chemical and biological entities for therapeutic purposes and/or contrast agents for medical imaging [229,334,335], as active components of biochips or biosensors [336], building blocks of in-situ forming scaffolds for tissue engineering [337,338], cell culture systems [339,340], and antimicrobial coatings [341,342], and as components of wound dressing formulations [343,344]. Other noticeable applications of nanogels in related fields include iron chelation therapy [345], biochemical separation and contaminant removal [346,347], and bio-catalysis [348]. 

In drug delivery, nanogels can offer some distinct advantages compared with other nano-constructs due to their inherent features, such as high colloidal stability in aqueous media and potentially also in blood, reduced adsorption of plasma proteins and hence prolonged circulatory half-life, flexibility, and possibility to take lateral drift velocity components when moving with the blood stream favoring extravasation, tunable adhesiveness to epithelial and endothelial cells, sizable drug loading capacity with a variety of loading and controlled release mechanisms, amenability to be combined with lipid cores or shells (nanolipogels), or with inorganic nanoparticles to enable multiple functions, e.g., for chemo-photothermal or chemo-photodynamic cancer therapy, or for image-guided therapies [349].

Nanogel features such as size, morphology, and composition all impact on nanogels circulation residence time and influence their biodistribution and clearance profiles. While early clearance is not desirable, accumulation at the disease site, and degradation or elimination after the delivery function has been accomplished are highly desirable. Several targeting strategies have been proposed and evaluated, that also set requirements on each of the above features of the nanocarrier. The impact of size, surface charge density, and surface chemistry on biodistribution, accumulation at the target site and clearance, have been the topic of several review papers [349,350,351,352,353,354]. One important consideration that emerges is that even subtle changes in the nanogel network can impact the biodistribution and tumor accumulation [355] as well as their cellular internalization mechanism, degree of uptake and potential for toxicity [225,356].

Although more and more scientific papers describing new nanogel formulations, synthesis methodologies, and potential applications in the biomedical field are appearing, only very few nanogels have already been introduced in clinical trials [357]. Today’s research in nanogel design and development for biomedical applications mainly focuses on how to overcome of the restrictions imposed by cost for commercial scale production and on some medical requirements and technological issues for their full exploitation as new platforms in biomedicine.

As already discussed before, the main advantages of using high-energy irradiation to synthetize hydrogel nanoparticles for biomedical applications include minimal recourse to potentially toxic chemicals, simple production schemes, possibility of fine-tuning nanogel size, crosslinking density and functionality by a proper selection of irradiation conditions, polymer concentration, and composition of the atmosphere (N_2_, N_2_O, air), and the possibility to obtain simultaneous sterilization when the absorbed doses are within the sterilization dose range. One major limitation of nanogel radiation-synthesis is related to the fact that crosslinking cannot be the dominant process for a few classes of potentially interesting hydrophilic polymers, such as polysaccharides and polypeptides, that mainly degrade under irradiation, but would undergo appropriate biodegradation if used as main components of nanogels. Crosslinking for these polymers may become competitive only upon chemical modification to introduce short alkyl chains and/or by achieving significant local increases of their concentration [358].

## 5. Radiation Chemistry of Natural Polymers

There are a large number of natural polymers and to review the radiation chemistry of all of them is beyond the scope of this review. This review will focus on five classes of natural polymers, polysaccharides, lignin, natural rubber (cis-1,4 polyisoprene), proteins, and RNA/DNA.

### 5.1. Radiation Chemistry of Polysaccharides

**Figure 28:**
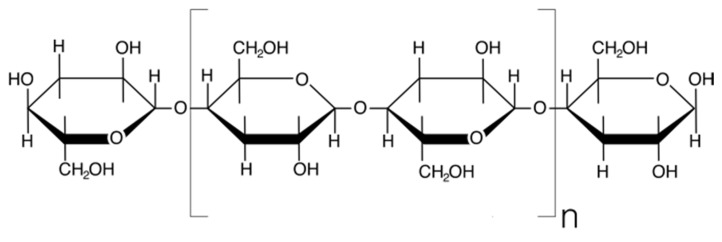
Chemical structure of the polysaccharide cellulose, note the glycosidic linkages between the glucose repeating units.

**Figure 29:**
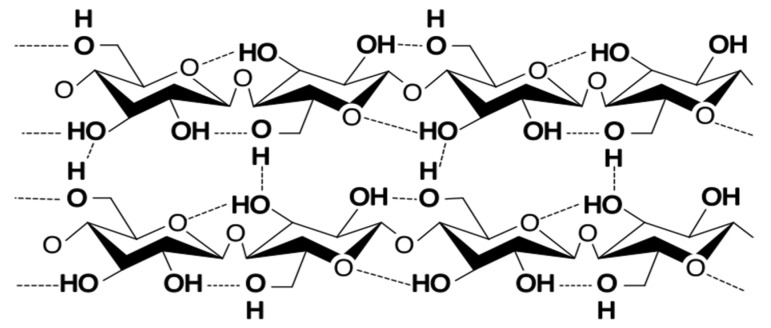
Crystalline structure of cellulose, note the inter and intra hydrogen bonding (dashed lines denote hydrogen bonds).

Polysaccharides are the most abundant natural polymers. They are long chains of carbohydrate (sugar) molecules, specifically polymeric carbohydrates are composed of monosaccharide units bound together by glycosidic linkages, as seen in Figure 28. Polysaccharides are widely distributed and are found in plant cell walls, seeds, and roots; algae; animals; bacteria; and fungi [359]. The most abundant monosaccharide in nature is the six-carbon sugar glucose and its derivatives as seen in cellulose and chitin. Polysaccharides can be amorphous as in hemicelluloses; crystalline such as cellulose and chitin and semicrystalline including amylopectin. Amorphous polysaccharides are most often highly branched polymers with linear polymers being more crystalline. The amount of crystallinity has been shown to affect the radiation chemistry of polysaccharides [360]. The type of glycosidic linkage can have a major effect on the physical and chemical properties of polysaccharides. Cellulose and amylose are both polymers of glucose, (1→4) linked d-glucose units. The difference is that cellulose is β (1→4) linked d-glucose units and amylose is α (1→4) linked d-glucose units. While both cellulose and amylose are insoluble in cold water amylose is soluble in hot water and cellulose is not [359]. This is attributed to the higher amount of hydrogen bonding, both inter and intra, in cellulose, Figure 29. Polysaccharides can also be made up of more than one saccharide. Alginate is a linear copolymer of β-(1–4) linked d-mannuronic acid and β-(1–4)-linked l-guluronic acid units [359]. There are many review articles and books on polysaccharides for additional information see [361,362,363].

### 5.2. Sold State Irradiation

When polysaccharides are irradiated in the solid state the energy is absorbed directly by the polymer and radicals are formed. This type of interaction is called a direct effect. When a polymer is irradiated two reactions can take place chain scission and crosslinking, with one predominating. In the case of polysaccharides chain scission predominates. In polysaccharides the absorbed energy causes the breakage of the glycosidic bond leading to a reduction of the molecular mass of the polymer [270]. After chain scission there are still free radicals present which can lead to ring opening of sugar units. These radicals can also lead to additional fragmentation of the polymer chain. Many studies have been conducted on the irradiation of natural polysaccharides and their derivatives in the sold state including cellulose and its derivatives [360,364,365,366,367,368,369,370,371,372,373,374,375,376,377,378,379,380,381], chitin and chitosan [382,383,384,385,386,387,388], starch [389,390], alginate [391,392], pectin [393,394], and dextran [395,396] to name a few. It should be noted that polysaccharides are hydroscopic and absorbed moisture can affect the radiation chemistry [397]. 

### 5.3. Aqueous Irradiation

In the case of a dilute aqueous solution of polysaccharides the energy will most likely be absorbed by the water producing the primary radiolysis products of hydrated electrons (e_aq_), hydrogen atoms (^•^H) and hydroxyl radicals (^•^OH). These primary radiolysis products can produce secondary products including hydrogen peroxide, perhydroxyl radical, and super oxide [398]. The most reactive of the primary radiolysis products towards polysaccharides is the hydroxyl radical. The hydroxyl radical randomly extracts hydrogen atoms from carbon hydrogen bonds. In polysaccharides the extraction is not selective, with all carbon-bound hydrogens equally likely to be extracted. The rate constant for the reaction of hydrogen atom is about an order of magnitude lower than for ^•^OH [398]. As with ^•^OH, ^•^H extracts hydrogen randomly from carbon hydrogen bonds. The reaction rate of hydrated electrons towards is at least two orders of magnitude lower than for ^•^OH. This is due to the fact that while e_aq_ react quickly with carbonyl groups they are slow to react with ethers and alcohol groups [399]. The extraction of hydrogens by ^•^H and ^•^OH produce radicals that lead to ring opening and chain scission [399]. There are many articles on the irradiation of polysaccharides in solution [400,401,402,403,404,405,406,407].

### 5.4. The Effect of Oxygen

The above discussion assumed the irradiations were conducted in an oxygen-free environment. In the presence of oxygen, carbon-centered radicals can react with oxygen to produce peroxyl radicals. As with carbon-centered radicals, peroxyl radicals can lead to ring opening, chain scission and stable products most often containing carbonyl groups [270]. Unlike many polymers, which tend to degrade much more in the presence of oxygen, polysaccharides degrade both in the presence of oxygen and without. In fact, some studies show that oxygen may reduce that amount of degradation in polysaccharides [359].

### 5.5. Radiation Crosslinking of Polysaccharides

While polysaccharides most often degrade upon irradiation, crosslinking can be accomplished under the right conditions. In concentrated solutions, cellulose derivatives have been shown to crosslink. This crosslinking is dependent on degree and type of substitution and at higher dose hydrogels can be formed [375,376,377,408,409]. Other polysaccharides have also been studied including substituted chitin and chitosan, and gum arabic [382,410,411].

Polysaccharides can also be crosslinked in solution and in the solid state in the presence of an acetylene gas. Carboxymethyl cellulose, dextran, pullulan, gum arabic and others have been studied [270,412,413,414,415]. Crosslinking is dependent on the degree of chain branching and degree and type of substitution [270]. At higher doses hydrogels can be formed.

### 5.6. Radical Lifetime in Polysaccharides

Polysaccharide radicals in solution decay very quickly when irradiation is suspended. Thus, there is no post irradiation effect. When crystalline and semicrystalline polysaccharides are irradiated in the solid-state radicals can be trapped in the crystalline structure after irradiation has stopped. These trapped radicals can slowly migrate to the amorphous region of the polymer where they undergo reactions including ring opening and chain scission [416,417,418,419,420,421]. Trapped radical can remain in the crystalline region for months. When processing crystalline polysaccharides, or other polymers, in the solid-state, post irradiation effects must be understood.

### 5.7. Radiation Chemistry of Lignin:

**Figure 30:**
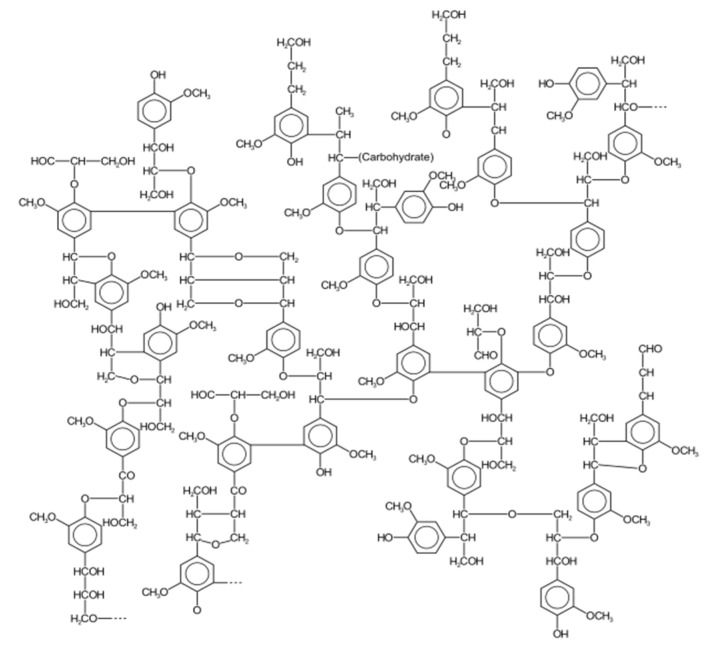
Model structure of lignin [428].

Lignin is a group of amorphous polyphenolic polymers that are produced in vascular plants and some algae. The structure of lignin is unknown, but it is believed that there are a number of different lignin structures. While the true structure(s) of lignin is not known, a model structure is illustrated in Figure 30 [422]. When lignin or model compounds are irradiated, phenoxyl and peroxyl radicals are produced and the glass transition of lignin is increased [423,424,425,426,427]. While there appears to be some reactions in lignin upon irradiation work by LaVerne et al. [360] has shown that the hydrogen production G-value for lignin is an order of magnitude lower than cellulose. 

### 5.8. Radiation Chemistry of Natural Rubber

Natural rubber also known as latex, India rubber and Amazonian rubber is a polymer of cis-1,4-polyisoprene with a molecular mass range of 100,000 to 1,000,000 Daltons. The worldwide production of natural rubber in 2018 was about 13.9 million metric tons. For most commercial use the rubber needs to be vulcanized (crosslinked). The main reaction when natural rubber is irradiated is crosslinking. In 1933 a patent was issued for the radiation crosslinking of rubber [429]. One of the main advantages of radiation crosslinking is that it is conducted at room temperatures which prevents thermal degradation. Many studies have been published on the crosslinking of rubber and rubber blends [430,431,432,433,434,435,436,437,438,439,440,441]. Blending rubber with other polymers changes the physical and chemical properties of the final polymer. While rubber can be crosslinked without the use of accelerator the dose required is high thus an accelerator is used for economic purposes.

### 5.9. Radiation Chemistry of Peptides and Proteins

Proteins are the most abundant organic compounds in animals. They are macromolecules of amino acids linked together by a peptide bond between the C1 carbon of one amino acid and the N2 nitrogen of the other. Molecules of less than 50 amino acids are called peptides while greater than 50 are proteins. In the solid or frozen-in-solution irradiation of proteins, only direct effects occur. This leads to random bond breakage resulting in fragmentation, backbone and disulfide bond breakage, and loss of helicity [442,443,444,445,446,447,448]. In aqueous solutions the most abundant reactions are due to primary radiation products of water. The hydroxyl radical is believed to be the most reactive primary product towards proteins. The radical will extract hydrogens from any accessible part of the protein with the backbone peptide and thiol hydrogens the most susceptible. Side change amino acids can also be attached with aromatic side chains more reactive than aliphatic [442,449,450,451,452,453]. More recently methionine oxidation by HO, H, e_aq_ and H_2_O_2_ has been studied [454,455]. The radiation effects of the primary radiation products of water are much greater than those of direct effects [442].

### 5.10. Radiation Chemistry of RNA and DNA

It has been very well known that ionizing radiation induces damage to biological systems. The mechanisms of radiation-induced damage are very complicated and vary tremendously from biological system to another. For example, the mechanisms involved from the radiation effects on DNA and RNA, proteins, fatty acids, and carbohydrates are different. The mechanisms of the radiation-induced damages strongly depend on the type of irradiation (high LET such as protons and alpha particles versus low LET such as gamma rays, X-rays, and electrons), dose and dose-rate, the presence of the molecular oxygen, the presence of antioxidants, and the presence of water.

Since most biological organisms contain a large amount of water, the research on radiation effects on biological systems has been concentrated in the presence of H_2_O. Under these conditions, most of the radiation effects are indirect, caused by the radiolysis of H_2_O and the radiolytically produced ^•^OH, e_aq_^−^, and H-atoms. These reactive species attack the DNA and RNA molecules at a diffusion controlled limit with reaction rate constants 10^8^–10^9^ L mol^−1^s^−1^. The radiolytically produced free radicals (DNA^•^ and RNA^•^), DNA and RNA cations (DNA^+^ and RNA^+^), and DNA and RNA anions (DNA^•−^ and RNA^•−^) undergo various complicated mechanisms leading to deadly damages such as single and double strand breaks and base and phosphate release. These damages are enhanced tremendously by the presence of O_2_. DNA^•^ and RNA^•^ radicals react with O_2_ to produce the corresponding peroxyl radicals; DNAO_2_^•^ and RNAO_2_^•^. These peroxyl radicals are very active and undergo various reactions, such as O_2_^•−^-elimination, and formation or decay of tetraoxides (DNAOO-OODNA and RNAOO-OORNA) leading to more damages. Similarly, the results of attacking the protein molecules by ^•^OH, e_aq_^−^, and H-atoms, from radiolysis of H_2_O, lead to the amine-release and damages. As for the carbohydrates, the ^•^OH radicals attack the glycosidic bonds leading to direct sessions on the backbone of the molecules. Finally, the fatty acids and liposomes are very prone to ionizing radiation. The ^•^OH radicals abstract H atoms from the backbone of the fatty acids or liposomes producing C-centered free radicals. These free radicals react with O_2_ to produce the corresponding peroxyl radicals (RO_2_^•^). The RO_2_^•^ abstract H atoms from another fatty acid (or liposome) producing another free radical and initiating a chain reaction. 

In the absence of H_2_O, the ionizing radiation interacts directly and rapidly with the biological molecules producing intermediates such as excited molecules, radicals, cations, and anions. These intermediates have a short life in the microsecond timescale or even less. Again, depending on the presence and absence of oxygen, the presence of traces amounts of water, the dose, and the dose-rate, these intermediates undergo various reactions. Similar to the indirect interaction (in the presence of H_2_O), radiolysis of DNA and RNA lead to single and double strand breaks, and base and phosphate release. Due to very strong hydrogen bonding, even very dry samples of DNA and RNA contain traces of water, which can contribute to indirect effects on the radiolysis of DNA and RNA. Although very little work has been published on the direct effects on proteins, fatty acids, and liposomes, most of the published results show that the direct ionizing radiation has the same effects as the indirect interactions. In both cases, the radiolytic products are amine-release, and oxidation in the proteins, and fatty acids and liposomes, respectively.

## 6. Summary

This survey emphasizes the evolution of research on radiation-initiated polymerization. Early investigations on radiation-initiated polymerization were mainly driven by the need for basic knowledge in this new field at the interface between polymer science and radiation chemistry, and by the perspective to develop processes competing with conventional methods for polymer production. Current activities are now oriented toward the study of radiation-induced processes of more complex chemical blends and/or in more complex media, using the recent developments for radiation sources and analytical methods. Besides the now well-established use of EB-curing in graphic arts, adhesives, and coatings, a variety of new applications are envisioned for advanced structural materials and for the production of key technological components for the healthcare and energy sectors. Promising prospects are under evaluation.

The use of radiation as an initiation source in controlled free radical graft copolymerization seems to open the door to designing new tunable surfaces in a controlled manner. The graft copolymers thus prepared may also lead to more complex structures via further chain growth or block extension by subsequent addition of monomer due to postpolymerization activity of their chain ends. Radiation-induced RAFT-mediated graft polymerization seems to be a very powerful technique for the achievement of tailored polymeric surfaces with well-defined properties.

Nanogels have been studied as potential carriers for the delivery of genes, proteins, and drugs; use in tissue engineering; as biosensors; and other materials. Since most of these applications are biomedical applications being able to synthesis nanogel without the need for toxic chemicals is important. The use of ionizing radiation for the crosslinking of the nanogels reduces or eliminates the need for toxic chemicals.

With more and more interest in the use of natural materials and green chemistry understanding how to control the effects of ionizing radiation on natural polymers is important.

## Figures and Tables

**Table 1:** g-values of common radiation-induced radical species in PVDF.

Radical	–CH_2_–CFOO^.^–CH_2_–	–CF_2_–C·H–CF_2_	–CF_2_–C·H_2_
g−value	$g_{\left|\right|}=2.0327$, $g_{⊥}=2.009$	$g_{iso}=2.004$	$g_{iso}=2.009$
$\Delta B_{pp}\left[Gauss\right]$	$\Delta B_{pp}^{\left|\right|}=20$, $\Delta B_{pp}^{⊥}=18$	$\Delta B_{pp}=33$	$\Delta B_{pp}=12$
A[Gauss]	-	$A_{F}=43$, $A_{H}=23$	$A_{H}=16$
